# Reversible Bond Dynamics Enable Crystallinity‐Healed COF Membranes for Selective Ion Transport

**DOI:** 10.1002/smll.202513711

**Published:** 2026-02-20

**Authors:** Wenming Zhao, Jindi Yang, Zhuyuan Wang, Junyang Zhang, Hao Zhang, Ming Yong, Xin Sun, Kaijie Xu, Xiangkang Zeng, Xiwang Zhang

**Affiliations:** ^1^ UQ Dow Centre For Sustainable Engineering Innovation School of Chemical Engineering The University of Queensland St Lucia Australia; ^2^ ARC Centre of Excellence for Green Electrochemical Transformation of Carbon Dioxide (GETCO2) The University of Queensland St Lucia Australia

**Keywords:** covalent organic frameworks, ion‐conducting membranes, post self‐healing

## Abstract

Covalent organic frameworks (COFs) offer ordered, nanometer‐scale channels with programmable chemistry and topology, making them promising membrane materials for selective ion transport. However, fabricating robust COF membranes that preserve high crystallinity remains a key challenge. Here we directly address this challenge by decoupling crystallization from membrane formation. COF membranes are first made by interfacial polymerization and subsequently healed under acid‐catalyzed hydrothermal conditions, which activate reversible COF linkage bond exchange and framework self‐correction. Using TpPa‐SO_3_H as a model, this healing process enhances the (100) x‐ray diffraction peak intensity by 25‐fold, resulting in a 375% increase in proton conductivity and enhanced monovalent cation‐cation selectivity. This “make‐then‐heal” strategy leverages dynamic covalent chemistry to produce structurally precise, crystallinity‐healed COF membranes.

## Graphical abstract

Conventional polymeric membranes are constrained by intrinsic trade‐offs between high ion permeability and precise selectivity due to disordered transport pathways. This article introduces a make‐then‐heal strategy for covalent organic framework membranes, decoupling crystallization from membrane formation. Post‐synthetic healing triggers framework self‐correction and produces highly ordered nanochannels for efficient and selective ion transport.

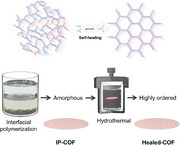

## 1 Introduction

**SCHEME 1:**
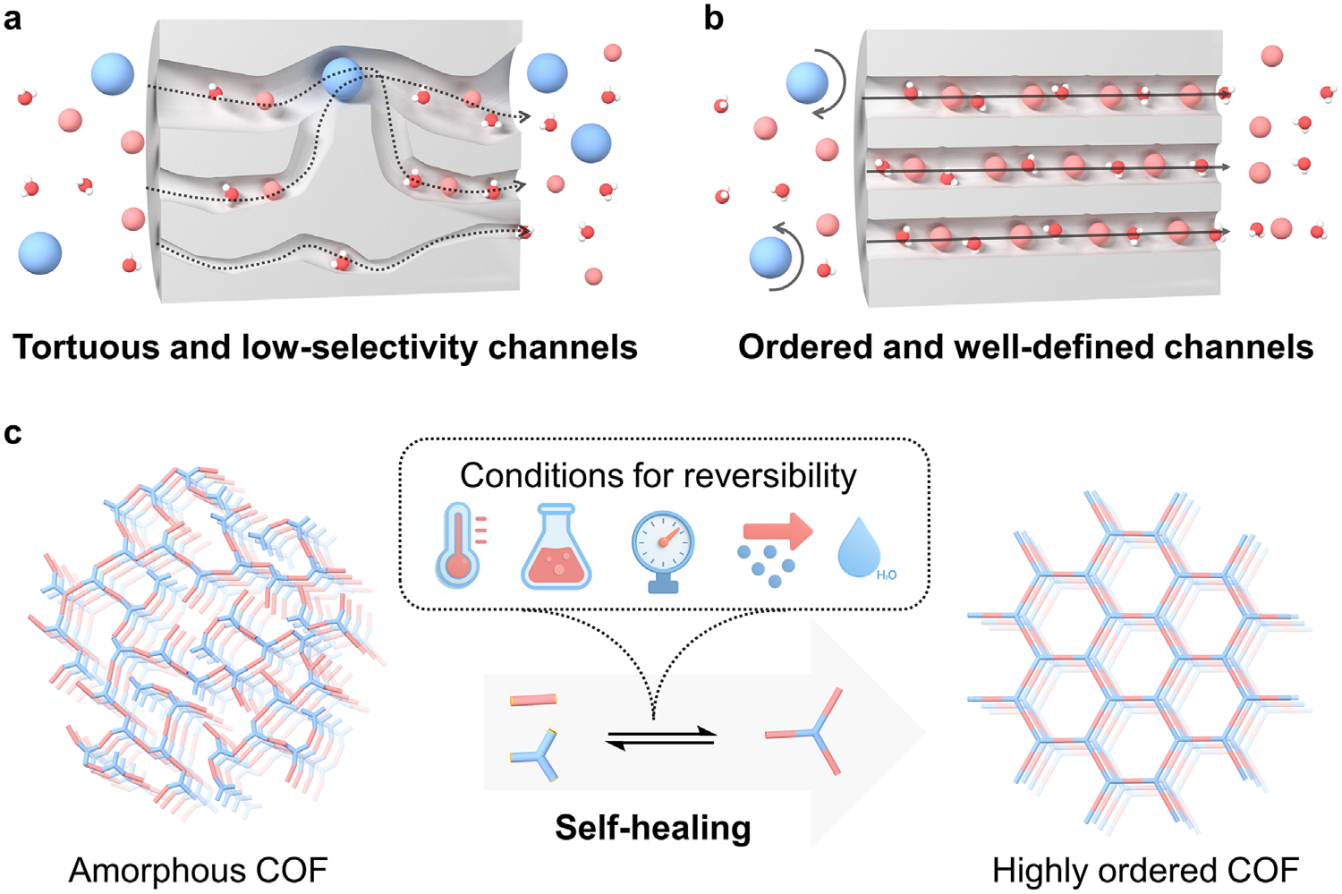
(a) Schematic illustration of tortuous and low‐selectivity transport pathways in conventional linear polymers, leading to poor molecular sieving performance. (b) Ordered and well‐defined channels in crystalline COFs, enabling efficient and selective transport. (c) Reversible transformation of an amorphous COF into a highly ordered COF under suitable external conditions (e.g., temperature, solvent, pressure, catalysts, or water), highlighting the self‐healing and error‐correction features of dynamic covalent chemistry.

Membranes with precisely controlled ion transport channels are fundamental to advancing separation technologies [1, 2, 3] and electrochemical devices [4, 5, 6, 7]. Conventional polymer‐based membranes typically rely on disordered free‐volume distributions and segmental dynamics, which makes it inherently challenging to achieve both high ion flux and sharp ion–ion discrimination simultaneously. As a result, ion transport membranes generally face a fundamental trade‐off between ion permeability and ion selectivity [8, 9, 10] (Scheme 1a).

When channel sizes approach the dimensions of an ion's hydration shell, transport behaviour becomes governed by the intricate interplay of size‐based exclusion [11], electrostatic interactions [12], and solvation energetics [13, 14]. In principle, well‐designed nano‐channels can reconcile high ionic conductivity with exceptional selectivity. Covalent organic frameworks (COFs) represent a promising class of crystalline, porous organic materials for engineering precise ion channels [15, 16, 17, 18, 19, 20]. These materials are constructed from multifunctional organic building blocks through reversible covalent bonds. This modular synthetic approach enables tuning pore topology and chemical environment, producing channels that couple steric exclusion with electrostatic selectivity [21, 22]. Importantly, the dynamic covalent bonds in COFs allow error correction during synthesis, in which reversible bond formation drives the framework toward more thermodynamically stable, long‐range ordered structures [23]. Such dynamic assembly should yield uniform nanometre‐scale channels capable of supporting both efficient ion transport and sharp ion–ion discrimination [24] (Scheme 1b).

Despite these compelling advantages, fabricating robust COF membranes while preserving high crystallinity remains a challenge. Top‐down approaches typically begin with pre‐synthesized COF crystals under solvothermal conditions, which are subsequently processed into membranes through exfoliation, filtration, or lamination techniques [25]. While such approaches retain COF crystallinity, the nanoscale assembly remains complex and introduces intra‐crystalline boundaries. These weak inter‐domain connections ultimately compromise the structural robustness and long‐term stability of the resulting membranes [3, 25, 26, 27]. More critically, such boundaries disrupt continuous ion‐transport pathways, obscure the intrinsic size and charge sieving mechanisms of COFs, and often lead to unstable ion conductivity and reduced ion selectivity. Conversely, bottom‐up strategies such as interfacial polymerization, or in situ polymerization can produce continuous and robust COF membranes in a single step [28, 29]. However, these rapid polymerization processes are inherently kinetically controlled and tend to suppress the reversible bond exchange essential for crystallization, resulting in frameworks with compromised long‐range order that appear polycrystalline or amorphous [24, 30, 31, 32]. The resulting structural disorder gives rise to ill‐defined nanochannels, which limits precise ion discrimination and often constrains ion conductivity despite good mechanical integrity. This intrinsic tension between maintaining structural fidelity for selective ion transport and achieving membrane robustness and continuity represents a fundamental challenge in COF membrane development.

Herein, we propose a “make‐then‐heal” strategy in which crystallinity enhancement is decoupled from the initial membrane fabrication (Scheme 1c). In this approach, continuous COF membranes are first formed and then subjected to a post‐synthetic treatment that activates the reversibility of dynamic covalent linkages. This activation allows frameworks to reorganize toward a more thermodynamically ordered state, thereby enhancing crystallinity. As exemplified by the TpPa‐SO_3_H COF membrane, we experimentally implement the “make‐then‐heal” concept.

## 2 Result and Discussion

**FIGURE 1:**
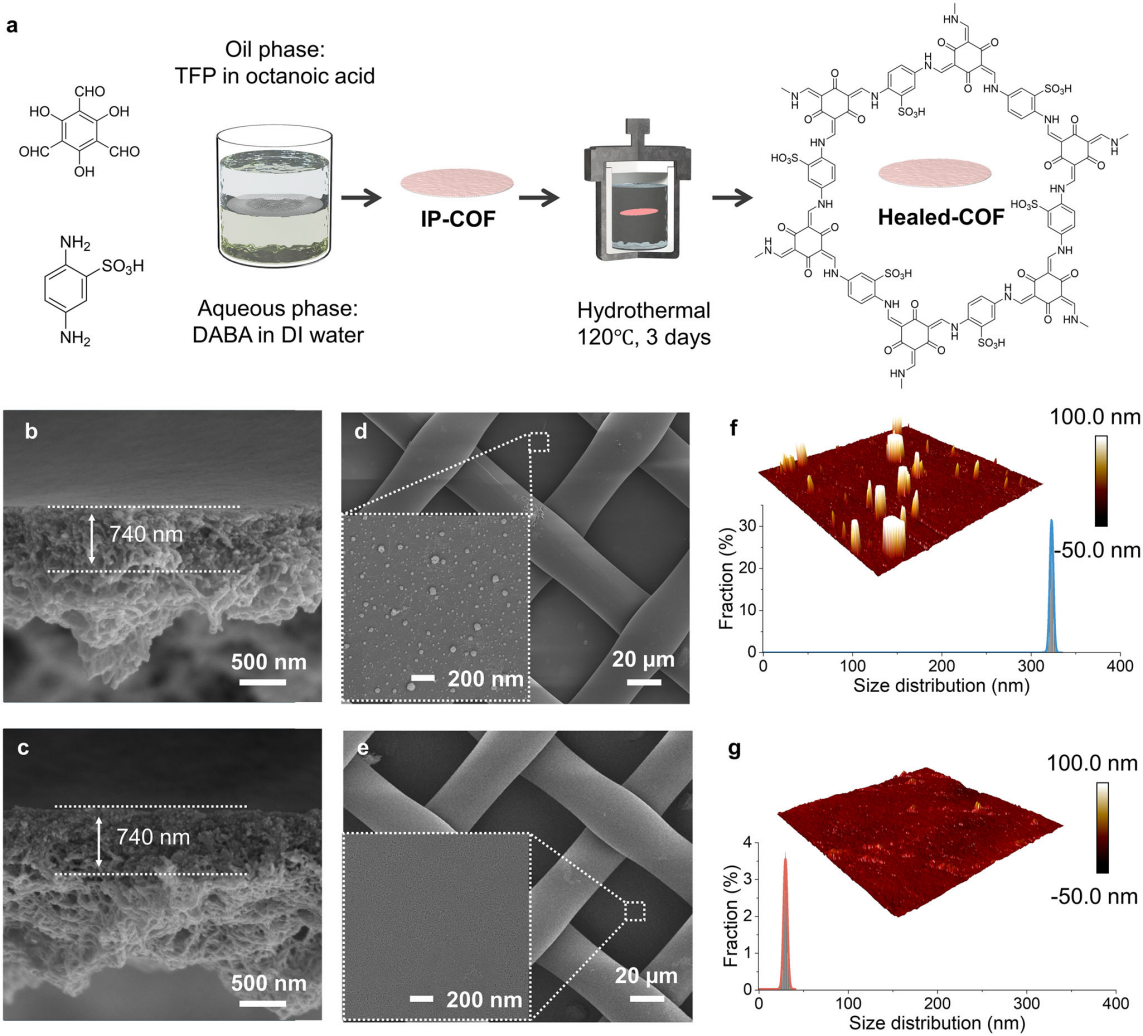
(a) Schematic illustration of the interfacial polymerization and healing process for COF membranes. Cross‐sectional SEM images of (b) IP‐COF and (c) Healed‐COF membranes. Surface SEM images of (d) IP‐COF and (e) Healed‐COF. AFM topography and size distribution of (f) IP‐COF and (g) Healed‐COF surfaces.

TpPa‐SO_3_H COF membrane was first fabricated by interfacial polymerization (IP) [29, 33], which was followed by a “healing” treatment to improve its crystallinity (Figure 1a). The IP process using 2,5‐diaminobenzenesulfonic acid (Pa‐SO_3_H) in water and 2,4,6‐triformylphloroglucinol (Tp) in an organic phase, with a nylon support yielded robust membranes (IP‐COF) under ambient conditions, but the rapid Schiff‐base condensation led to a mostly amorphous framework [23, 34, 35]. To overcome this limitation, the membranes were subjected to a hydrothermal healing step in which acetic acid catalysed imine bond exchange, while elevated temperature and solvent mobility facilitated lattice reorganization [23, 36, 37]. After being reacted at 120°C for 72 h, this post‐synthetic treatment yielded structurally refined membranes (Healed‐COF).

As shown in Figure S1, both IP‐COF and Healed‐COF remained macroscopically intact and defect‐free. Moreover, with the nylon support, the Healed‐COF membrane exhibited decent mechanical robustness (Figure S6). Cross‐sectional SEM images confirmed that the Healed‐COF membrane retains the asymmetric architecture with ∼740 nm thickness of the pristine IP‐COF membrane (Figure 1b,c). A dense, smooth skin layer forms on the organic‐facing side, while a looser fibrous sublayer develops on the aqueous‐facing side. This asymmetric architecture arises directly from the IP conditions [33]. During the IP process, strong solvation of the ionic TpPa‐SO_3_H oligomers in the water phase creates a porous, fibrous network, whereas limited compatibility with the organic phase yields a smooth, compact skin.

While the bulk structure remains unchanged, surface analyses reveal that healing refines the membrane morphology. SEM images of the top surface show that the Healed‐COF develops a smoother and more uniform skin layer compared to the particulate, rough texture of the IP‐COF membrane (Figure 1d,e). The fibrous sublayer also becomes more regular after healing, though its overall porosity is maintained (Figures S2 and S3). Atomic force microscopy (AFM) quantifies these changes. The IP‐COF skin consists of ∼300–350 nm particulate domains with a root‐mean‐square roughness (Rq) of 22.6 nm (Figure 1f). After healing, the surface domain size shrinks below 50 nm and Rq drops six‐fold to 3.8 nm (Figure 1g), indicating that larger amorphous clusters have reorganized into a finer and more homogeneous texture. Similarly, on the fibrous side, the roughness decreases from Rq ≈157 to117 nm, and the feature size distribution narrows (Figures S4 and S5).

**FIGURE 2:**
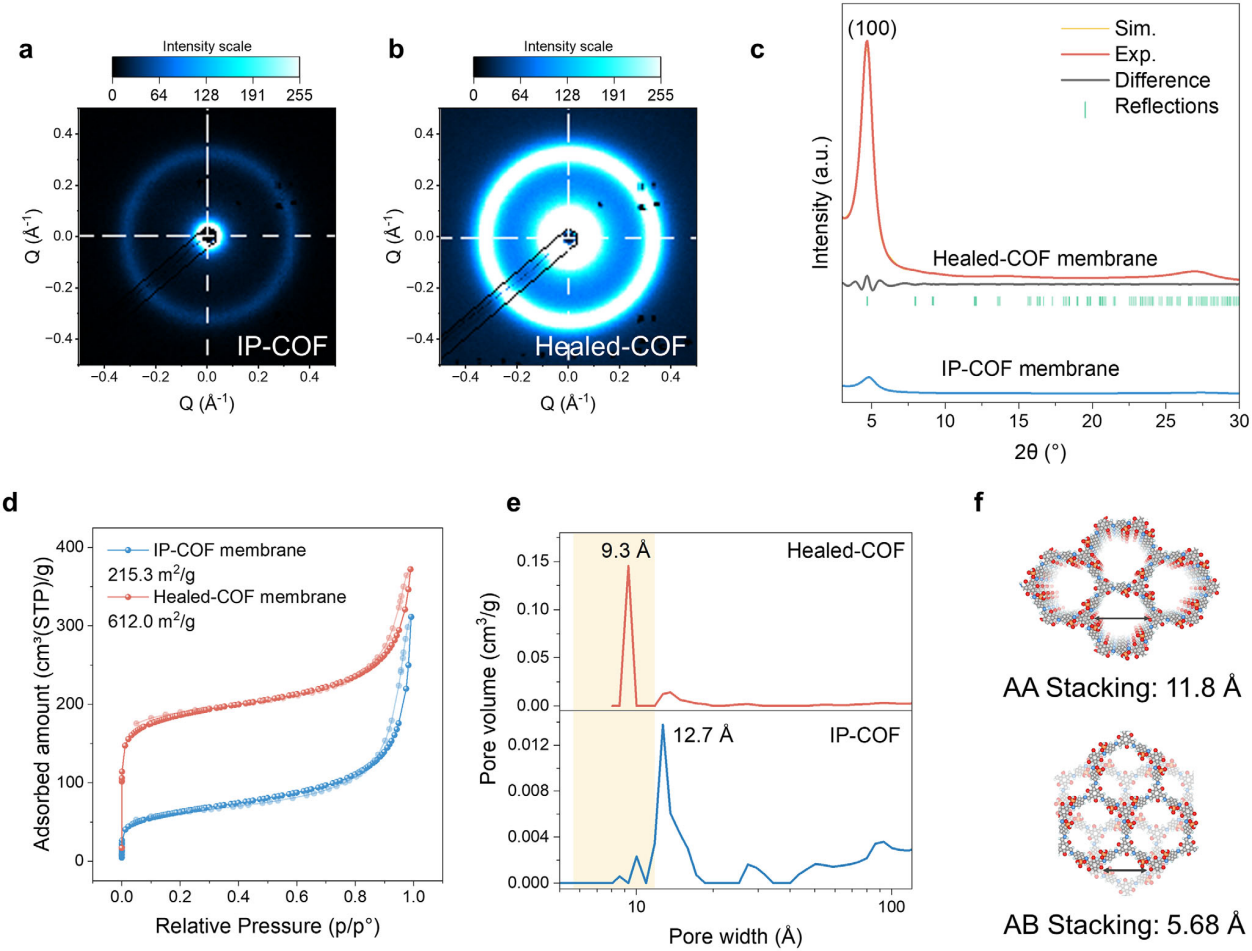
GIXRD transmission spectra of (a) IP‐COF and (b) Healed‐COF membranes. (c) GIXRD patterns showing a distinct peak at 2θ ≈ 4.7°, indexed to the (100) plane. (d) N_2_ adsorption–desorption isotherms at 77 K of IP‐COF and Healed‐COF membranes. (e) Pore size distributions derived from adsorption data, with the shaded region indicating the theoretical pore size range based on AA and AB stacking models. (f) Structural representations of AA and AB stacking.

Scattering and diffraction analyses confirm that healing enhances framework order and channel uniformity. As shown in Figure 2a,b, GIXRD transmission spectra of the as‐prepared IP‐COF display only weak, broad features, indicating a low degree of long‐range order and limited crystallinity. Whereas the Healed‐COF exhibits sharp, intense reflections, most prominently the (100) peak at 2θ ≈ 4.7° [38, 39], suggesting a pronounced enhancement in structural ordering after the healing process. In the grazing‐incidence geometry (Figure 2a,b), where the X‐ray beam is parallel to the membrane cross section, the diffraction pattern appears as a continuous ring without pronounced azimuthal intensity modulation, indicating a high degree of structural homogeneity across the membrane thickness.

To validate the structural assignment, experimental XRD of the Healed‐COF was compared with a simulated pattern derived from a TpPa‐SO_3_H COF model refined by the Pawley method, showing excellent agreement (Figure 2c). This agreement confirms that the healed membrane preserves the expected lattice symmetry and framework topology of the designed COF structure. A schematic of the structural model is provided in Figure S7.

A schematic of the structural model is provided in Figure S7

Quantitative analysis reveals a ∼5.6‐fold in‐plane and ∼6.0‐fold out‐of‐plane increase in surface GISAXS intensity (Figures S8–S10), reflecting anisotropic growth and enhanced crystallinity within the membrane, together with a ∼24.9‐fold enhancement in bulk XRD intensity (Figure 2c). Importantly, the increased diffraction intensity arises from framework reorganization rather than the removal of unreacted monomer or soluble oligomers. Treatment of membranes in a mixed solvent (Dioxane and mesitylene, 4:1 v/v) [40, 41] that fully solubilises the monomers at 120°C for 72 h caused no discernible change in x‐ray diffraction, indicating that thermal‐solvent exposure alone does not induce the enhanced long‐range ordering (Figure S11). Moreover, healing experiments conducted on membranes treated with different acetic acid concentrations show that the peak intensity increases from the IP ‐COF membrane to the DI water–treated membrane and further to the 1 m acetic acid–treated membrane, followed by a decrease for the 6 m acetic acid–treated membrane (Figure S12). This decrease at high acid concentration is likely attributable to excessive hydrolysis induced by over‐catalysis.

Nitrogen sorption‐desorption further validates the structural refinement achieved by healing. The as‐prepared IP‐COF membrane exhibits a type II isotherm with a low BET surface area (∼215 m^2^g^−1^) (Figure 2d), reflecting poorly developed microporosity with blocked or irregular channels. The Healed‐COF membrane shows a type I isotherm with steep uptake at low relative pressure and a tripled surface area (∼612 m^2^g^−1^), indicative of an open, interconnected micropore framework. The pore‐size distribution narrows and shifts into the regime expected for an ordered TpPa‐SO_3_H lattice (Figure 2e). After healing, broad, ill‐defined pores in IP‐COF membrane are transformed into ∼1 nm channels, consistent with theoretical values based on stacking models (AA stacking: 11.8 Å, Figure S13; AB stacking: 5.68 Å, Figures S14; 2f).

Spectroscopic evidence confirms that this improvement in order and porosity is achieved without altering the chemical identity of the framework. FTIR spectra of the IP‐COF and Healed‐COF are virtually identical (Figure S15). Both show the characteristic vibrational bands of the TpPa‐SO_3_H keto‐enamine linkages, including C═C stretching at 1569 cm^−1^, the sulfonate S═O stretch at 1427 cm^−1^, and C─N stretching at 1248 cm^−1^, with no new peaks appearing after healing. Likewise, X‐ray photoelectron spectra remain unchanged (Figure S16). The C 1s exhibits peaks for C─C/C═C (∼284.6 eV), C─N (∼285.6 eV), and C═O (∼288.2 eV), and the N 1s signal shows the same components for imine (C═N at ∼398.5 eV) and amine (C─N at ∼400.0 eV). No shifts or new features are observed. These findings confirm that the covalent framework is chemically intact. The improvement arises from reversible imine bond exchange and reorganization within the existing structure, rather than any new bond formation or irreversible hydrolysis.

**FIGURE 3:**
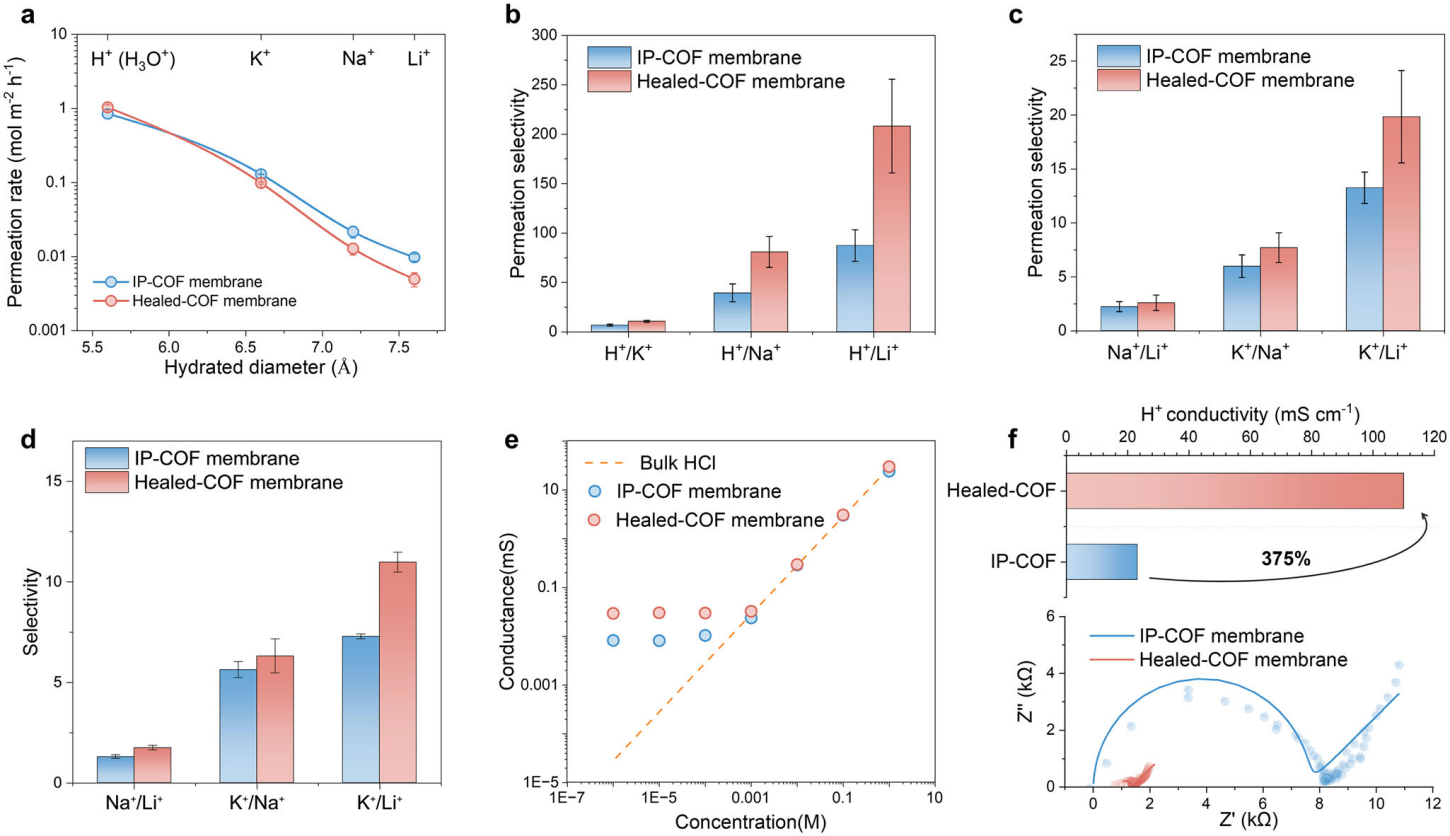
Ion transport performance of IP‐COF and Healed‐COF membranes. (a) Ion permeability of the IP‐COF and Healed‐COF membranes. Error bars in all panels represent the standard deviation of three parallel tests. (b) Permeation selectivity of the two membranes for H^+^ over alkali metal ions (H^+^/K^+^, H^+^/Na^+^, H^+^/Li^+^). (c) Permeation selectivity between alkali metal ions (Na^+^/Li^+^, K^+^/Na^+^, K^+^/Li^+^). (d) Selectivity of the membranes under applied voltage bias. (e) Conductance as a function of HCl concentration, showing a non‐linear decline compared with the bulk value (dashed line). (f) H^+^ conductivity of IP‐COF and Healed‐COF membranes.

The significance of improved channel order is ultimately reflected in practical separation performance. Ion permeation through both membranes follows a clear size‐dependent trend, with flux decreasing as hydrated diameter increases (H^+^ > K^+^ > Na^+^ > Li^+^) (Figure 3a). In addition, healing sharpens ion–ion discrimination. Under concentration gradients (Figure 3b), H^+^ selectivity over alkali cations rises dramatically: H^+^/K^+^, H^+^/Na^+^, and H^+^/Li^+^ increase 1.6‐, 2.1‐, and 2.4‐fold, reaching 10.5, 80.7, and 208.1, respectively. Importantly, these substantial gains are accompanied by increased proton flux (from 0.85 to 1.03 mol·m^−2^·h^−1^), confirming that healing strengthens proton‐exclusive pathways rather than imposing additional transport resistance. Selectivity improvements are also evident among the monovalent cations, with K^+^/Na^+^ and K^+^/Li^+^ rising from 5.97 to 7.69 and 13.25 to 19.84, respectively, corresponding to 1.3‐ and 1.5‐fold enhancements (Figure 3c).

Notably, the permeation rates of Li^+^, Na^+^, and K^+^ are lower in Healed‐COF than in IP‐COF. This difference arises from the improved structural integrity and enhanced continuity of the transport channels after healing, rather than from a change in the intrinsic transport mechanism. In Healed‐COF, the effective transport pathways are more continuous, narrowed, and regularized as indicated by Figure 2d,e, leading to the suppression of defect‐assisted, nonselective permeation routes that otherwise facilitate the diffusion of hydrated alkali‐metal ions, which require larger effective free volumes [42]. Meanwhile, the resulting ordered nanochannels provide a confined, hydrogen‐bond‐rich environment that sustains continuous proton‐hopping networks [43, 44], while imposing steric and energetic constraints on hydrated Li^+^/Na^+^/K^+^ permeation. As a result, healing‐induced pathway regularization reduces alkali‐metal ion permeation while favouring proton transport.

Bias‐driven transport provides a complementary perspective. Linear sweep voltammetry shows that Healed‐COF membrane conducts K^+^ more efficiently while attenuating Li^+^ transport relative to as‐prepared IP‐COF membrane (Figures S17 and S18), yielding field‐driven K^+^/Na^+^ and K^+^/Li^+^ selectivity of 6.3 and 11.0, compared with 5.6 and 7.3 prior to healing process (Figure 3d). These 1.1‐ and 1.5‐fold increases mirror the concentration‐gradient trends, indicating that healing improves continuity of ion‐conducting pathways and reduces defect‐mediated leakage.

To probe the proton transport mechanism, we measured the conductivity of IP‐COF and Healed‐COF membranes across HCl concentrations from 10^−6^ to 1.0 m. Both membranes exhibit characteristic features of fixed‐charge‐dominated channels (Figure 3e). A low‐concentration conductivity plateau arising from Donnan partitioning and electric‐double‐layer overlap, followed by crossover to bulk‐like transport above ∼10^−3^ m. Healing does not alter this electrostatic regime but markedly amplifies it. In the dilute region, healed membrane shows nearly threefold higher conductivity than IP‐COF membrane, while convergence to bulk values at high concentration remains unchanged. These results indicate that hydrothermal healing improves continuity of counter‐proton pathways and reduces defect‐mediated leakage without perturbing the intrinsic Donnan/EDL‐controlled transport mechanism.

To directly probe intrinsic proton conduction, four‐point EIS was performed under 100% relative humidity in the absence of external electrolyte. The Healed‐COF membrane achieved a conductivity of 110.03 mS·cm^−1^ compared to 23.14 mS·cm^−1^ for the IP‐COF, representing a 375% increase (Figure 3f). A comparison with the proton conductivities of literature‐reported membranes is provided in Table S1, and the membrane exhibits good overall performance. Consistent improvements across concentration‐ and field‐driven transport align with the structural evidence and indicate that hydrothermal healing produces more continuous and ordered ion transport pathways.

Ion exchange capacity measurements showed that the pristine IP‐COF and Healed‐COF membranes exhibit nearly identical IEC values (Figure S19), indicating that the density of proton‐exchange sites remains essentially unchanged after healing. In contrast, the water uptake of the Healed‐COF membrane increases to 337.2 mg g^−^^1^, about 1.44 times that of pristine IP‐COF (Figure S19). This enhanced water uptake suggests the formation of a more continuous hydrogen‐bonded water network within the membrane, facilitating more efficient Grotthuss‐type proton hopping and accounting for the observed increase in proton conductivity.

To further evaluate the long‐term operational stability of the Healed‐COF membrane, a continuous ion permeation test was performed for 240 h. As shown in Figure S20, the ion flux remains stable throughout the testing period, indicating good long‐term structural integrity and functional durability of the membrane.

## 3 Conclusion

In summary, we introduce a “make‐then‐heal” strategy that decouples membrane formation from crystallization in COF fabrication. Interfacial polymerization ensures the formation of continuous and mechanically robust membranes, while subsequent hydrothermal healing induces dynamic imine bond exchange, transforming initially amorphous frameworks into ordered crystalline structures. The healed COF membrane exhibit uniform and well‐defined nanochannels, enabling simultaneous enhancements in proton conductivity and ion selectivity, thereby effectively overcoming the long‐standing trade‐off commonly encountered in conventionally fabricated membranes. By exploiting the intrinsic reversibility of covalent bonding, the make‐then‐heal strategy shows promising applicability to other COF systems, advancing their design for molecular‐level ion transport and separation applications.

## Author Contributions

Conceptualization was done by X.Z., Z.W. and J.Y. The experiments were performed by W.Z. The manuscript was prepared by J.Y., W.Z., and Z.W. All authors participate in membrane characterizations, data analysis and validation.

## Conflicts of Interest

The authors declare no conflicts of interest.

## Supporting information




**Supporting File**: smll72912‐sup‐0001‐SuppMat.docx.

## Data Availability

The data pertaining to this study are either provided in the Article and Supplementary Files or can be obtained from the corresponding authors upon reasonable request.
