# Supplementary material for: Reversible Bond Dynamics Enable Crystallinity‐Healed COF Membranes for Selective Ion Transport
Source: Small. 2026 Feb 20;22(23):e13711. doi: 10.1002/smll.202513711 (PMC13100557; doi:10.1002/smll.202513711)
Supplement: Supplementary file 1 — Supporting File: smll72912‐sup‐0001‐SuppMat.docx. [file SMLL-22-e13711-s001.docx]

**Supplementary information**

Reversible Bond Dynamics Enable Crystallinity-Healed COF Membranes for Selective Ion Transport

Wenming Zhao^1†^, Jindi Yang^1, 2†^, Zhuyuan Wang^1, 2*^, Junyang Zhang^1^, Hao Zhang^1^, Ming Yong^1^, Xin Sun^1^, Kaijie Xu^1, 2^, Xiangkang Zeng^1, 2^, Xiwang Zhang^1,2*^

^1^UQ Dow Centre for Sustainable Engineering Innovation, School of Chemical Engineering, The University of Queensland, St Lucia, QLD 4072, Australia

^2^ARC Centre of Excellence for Green Electrochemical Transformation of Carbon Dioxide (GETCO2), The University of Queensland, Brisbane, QLD 4072, Australia

^*^Email: zhuyuan.wang@uq.edu.au, xiwang.zhang@uq.edu.au

^†^These authors contributed equally to this work

**1. Materials and chemicals**

2,4,6-triformylphloroglucinol (Tp) was purchased from Tokyo chemical industry Co., Ltd. (TCI), 2,5-diaminobenzenesulfonic acid (Pa-SO_3_H), n-octanoic acid, sodium formate, Mesitylene, Hexane, Ethanol, Acetic acid and hydrochloric acid were purchased from Merck (Sigma-Aldrich). Millipore nylon membrane filter, 60.0 μm Pore Size (diam. 47 mm, hydrophilic) was purchased from Merck (Sigma-Aldrich). Sodium chloride, potassium Chloride and Lithium Chloride were purchased from Sigma-Aldrich. All commercially available chemicals and solvents were used without further purification. Deionized water (>18 MΩ∙cm) was produced by Milli-Q Water System.

**2. Methods**

**Fabrication of the TpPa-SO_3_H ionic COF membrane**

The ionic COF membranes (iCOFMs) were prepared by a modified dual-activation interfacial polymerization^1^. An organic phase was prepared by dissolving the aldehyde monomer, 2,4,6‑triformylphloroglucinol (Tp, 0.334 mmol, 70.14 mg) inti a mixed solvent of n‑octanoic acid (25 mL) and mesitylene (8.33 mL). N‑octanoic acid served as an activator for the aldehyde monomer, whereas mesitylene acted as an inert co‑solvent. An aqueous phase was prepared by dissolving the amine monomer, 2,5‑diaminobenzenesulfonic acid (Pa‑SO₃H, 0.50 mmol, 93.99 mg) and sodium formate (0–1.75 mmol; 0–3.5 equiv relative to Pa‑SO₃H) into 50ml deionized water (DI water) with sonication for 20 min. Interfacial polymerization was conducted in a custom‑built cell comprising upper and lower compartments separated by rubber gaskets. The amine solution (50 mL) was poured into the bottom compartment, and a nylon membrane filter was gently placed on top of the aqueous phase to avoid bubble formation on either side of the filter. Subsequently, the top compartment was assembled, and Tp solution was added dropwise into the top compartment to form the organic layer. After standing undisturbed for 24 h, the resulting TpPa‑SO₃H iCOFM supported on the nylon filter was gently retrieved with tweezers. The membrane diameter was 4 cm, matching the diameter of the compartment opening. The iCOFM was rinsed sequentially with DMF, ethanol, and deionized water, and stored in water for subsequent characterization and performance evaluation^2^. Prior to dry-film characterization, as-prepared wet membranes were subjected to sequential solvent exchange: three washes with ethanol followed by three washes with n-hexane and then placed between two sheets of nonwoven fabric and air‑dried at room temperature to obtain a flat and intact membrane. Ethanol removes residual high-boiling polar synthesis solvents and loosely bound oligomers, while the final n-hexane exchanges replace the pore fluid with a low-surface-tension solvent to minimize capillary stresses during drying; this two-step activation preserves the intrinsic microporosity and prevents pore collapse that can arise from direct evaporation of polar solvents^3^.

**Post-treatment of the TpPa-SO_3_H iCOFMs**

Hydrothermal method was used to post-treat the iCOFM^4, 5^ ^6^ ^7^. 4 mL of acetic acid solution (1 mol L⁻¹) and 40 mL of deionized water were added to a 100 mL glass tube sealed with a PTFE screw cap. The mixture was sonicated for 10 min, and the TpPa-SO₃H iCOFM supported on a nylon substrate was gently immersed in the solution. The tube was then sealed and heated at 120 °C for 3 days. After post-treatment, the deep-red post-treated iCOFM was collected and washed three times with anhydrous ethanol and deionized water.

**Characterizations**

Grazing incidence X-ray diffraction (GIXRD) was performed on a Rigaku SmartLab system configured for in-plane measurements, utilizing a Cu Kα radiation source, operating at 45 kV and 200 mA at an incident angle of 0.5°. Grazing-incidence wide-angle X-ray scattering (GIWAXS) was used to probe the cross-sectional crystallographic structure of the membranes before and after reconstruction. Measurements were carried out at the SAXS/WAXS beamline of the Australian Synchrotron using 12 keV photons. The sample-to-detector distance was calibrated with a silver behenate standard. A data acquisition time of 30 s was used. The element composition was analyzed with X-ray photoelectron spectroscopy (XPS, Kratos Ultra) with a monochromatic Al Kα X-ray source. Fourier transform infrared (FT-IR) spectra were recorded on a IRTracer-100. AFM (Dimension Icon, Bruker Co. Ltd.) analysis was utilized to characterize the surface roughness of the membrane. The surface and cross-sectional morphologies of the membranes were observed using a field-emission scanning electron microscope (JEOL JSM-7800F, Japan) operated at 5 kV. Prior to imaging, all samples were coated with a thin layer of Platinum. Nitrogen sorption analysis was performed on a Micromeritics Flex at 77 K to obtain catalyst specific surface area using the Brunauer–Emmett–Teller (BET) method and pore size distribution using the DFT method. Catalysts were degassed under vacuum at 100 °C for 12 h before N₂ sorption measurements.

**Proton conductivity measurement**

The in-plane proton conductivity of iCOFMs was measured by electrochemical impedance spectroscopy (EIS) using a four-electrode configuration. Membrane samples were cut into rectangular strips and mounted in a custom cell equipped with platinum electrodes. The impedance spectra were collected on a Bio-Logic VSP-300 multichannel potentiostat (Bio-Logic Science Instruments, France) over the frequency range of 1 MHz to 1 Hz with an AC perturbation of 10 mV. The ohmic resistance was extracted from the high-frequency intercept of the Nyquist plot, and the proton conductivity (σ) was calculated according to:

$$\sigma= L / (R \times W \times t)$$

where *L* is the electrode spacing, *W* is the membrane width, *t* is the membrane thickness, and *R* is the measured resistance. All measurements were conducted under controlled humidity and temperature (25 °C, 100% RH).

**Ion permeation experiment**
Ion permeation through iCOFMs was evaluated using a custom-built H-cell. In a typical test, 20 mL of 0.1 M XCl (X = H⁺, K⁺, Na⁺, Li⁺) solution and deionized water were introduced into the feed and permeate chambers, respectively. Both chambers were magnetically stirred at 350 rpm to minimize concentration polarization. The conductivity of the permeate solution was monitored at room temperature with a LabCHEM conductivity–pH meter. The permeate salt concentration (*C*) was determined from the measured conductivity (κ) and the molar conductivity of the electrolyte (Λₘ) according to:

$$C=\frac{\kappa}{\Lambda m}$$

The ion permeation flux (*Jᵢ*, mmol h⁻¹ m⁻²) was then calculated by:

$$J_{i}=\frac{VC}{At}$$

where *V* is the permeate volume, *A* is the effective membrane area, and *t* is the permeation time.

**Ion current measurement**
Ion transport properties of iCOFMs were further examined by linear sweep voltammetry (LSV) in the same H-cell. Both compartments were filled with equal volumes of electrolyte solution, and Ag/AgCl reference electrodes were employed to apply a transmembrane potential. Current–voltage (I–V) curves were recorded using a Keithley 2450 source meter in the range of –1.5 to +1.5 V at a scan rate of 50 mV s⁻¹. Each measurement was repeated at least three times, and average current values were used for analysis.

**Structural modelling and computational characterization**

AA- and AB-stacked COF models were built in Materials Studio and geometry-optimized with the Forcite module using the Dreiding force field^8^. Atomic partial charges were approximated via charge equilibration (QEq)^9^. Powder X-ray diffraction (PXRD) patterns were simulated in Reflex, and Pawley refinements were performed over 2θ = 3–61° using a pseudo-Voigt profile. The refined parameters included unit-cell constants a, b, c, the FWHM parameters U, V, W, profile shape parameters NA, NB, and the zero-shift. The largest free sphere pore sizes, surface area and pore size distribution were calculated by Zeo++^10^ with a probe radius of 1.86 Å to simulate nitrogen adoption.

**3. Supplemental Figures**


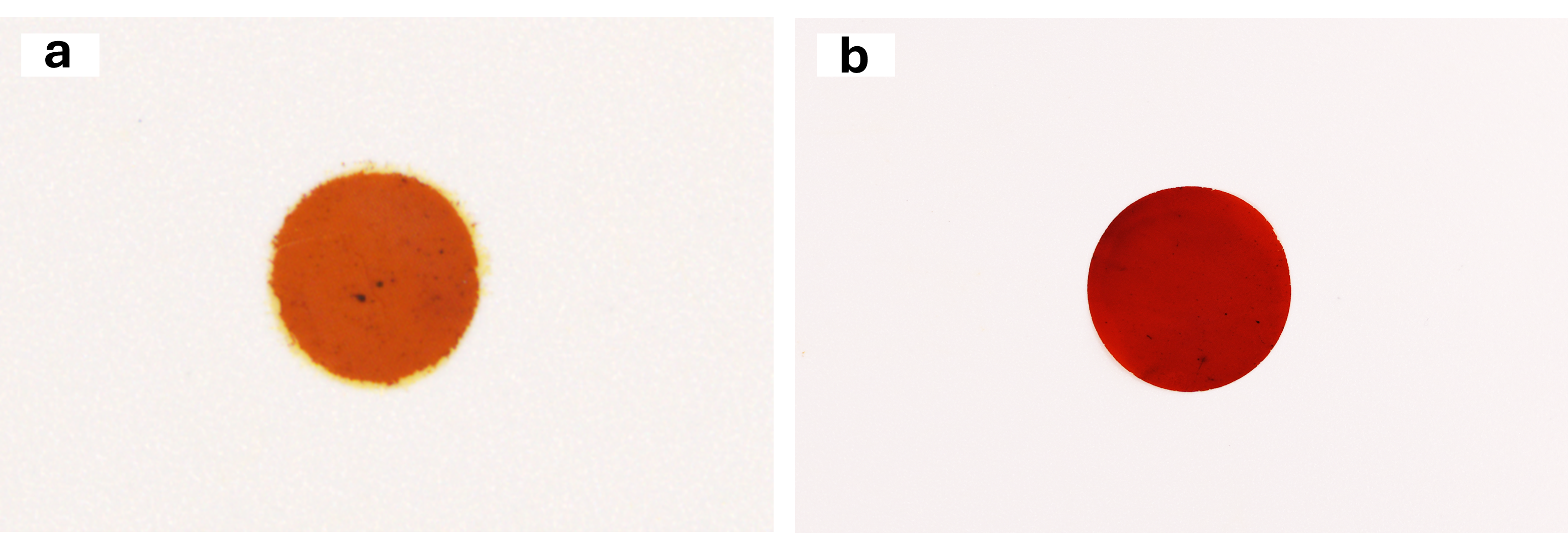


Figure S1. Digital photo of the IP-COF membrane (a) and Healed-COF membrane (b).


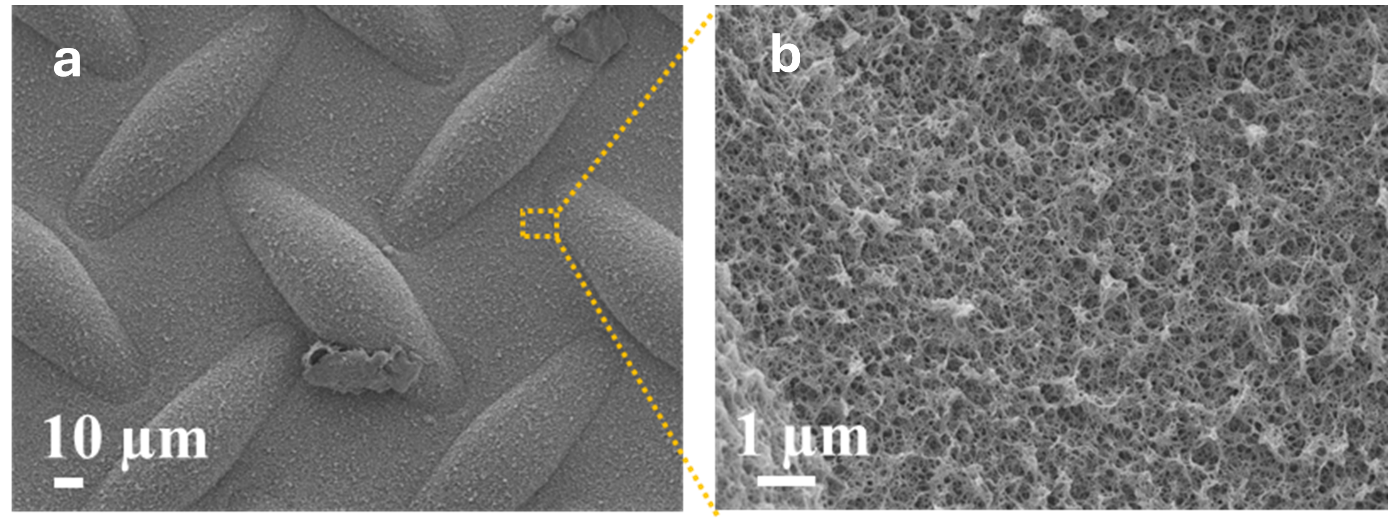


Figure S2. Surface SEM images of the bottom side of the IP-COF membrane. (a) surface morphology (scale bar: 10 µm) and (b) enlarged view showing the porous structure (scale bar: 1 µm).


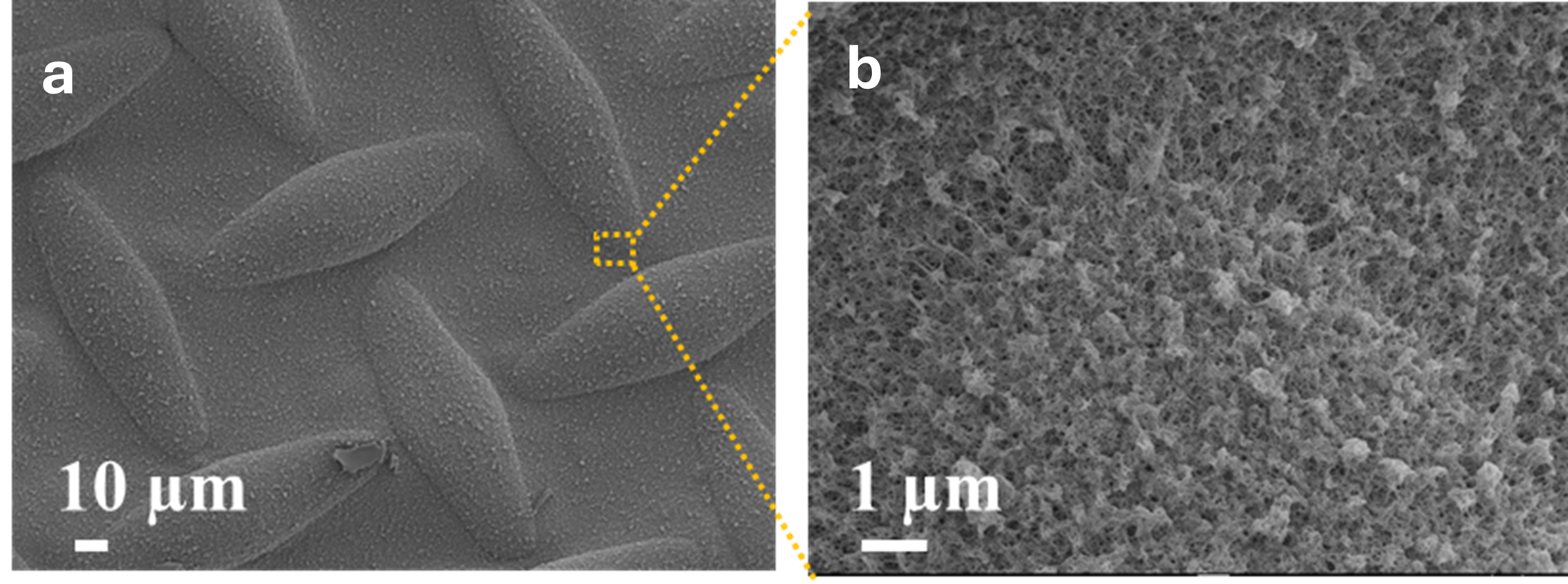


Figure S3. Surface SEM images of the bottom side of the Healed-COF membrane. (a) surface morphology (scale bar: 10 µm) and (b) enlarged view showing the porous structure (scale bar: 1 µm).


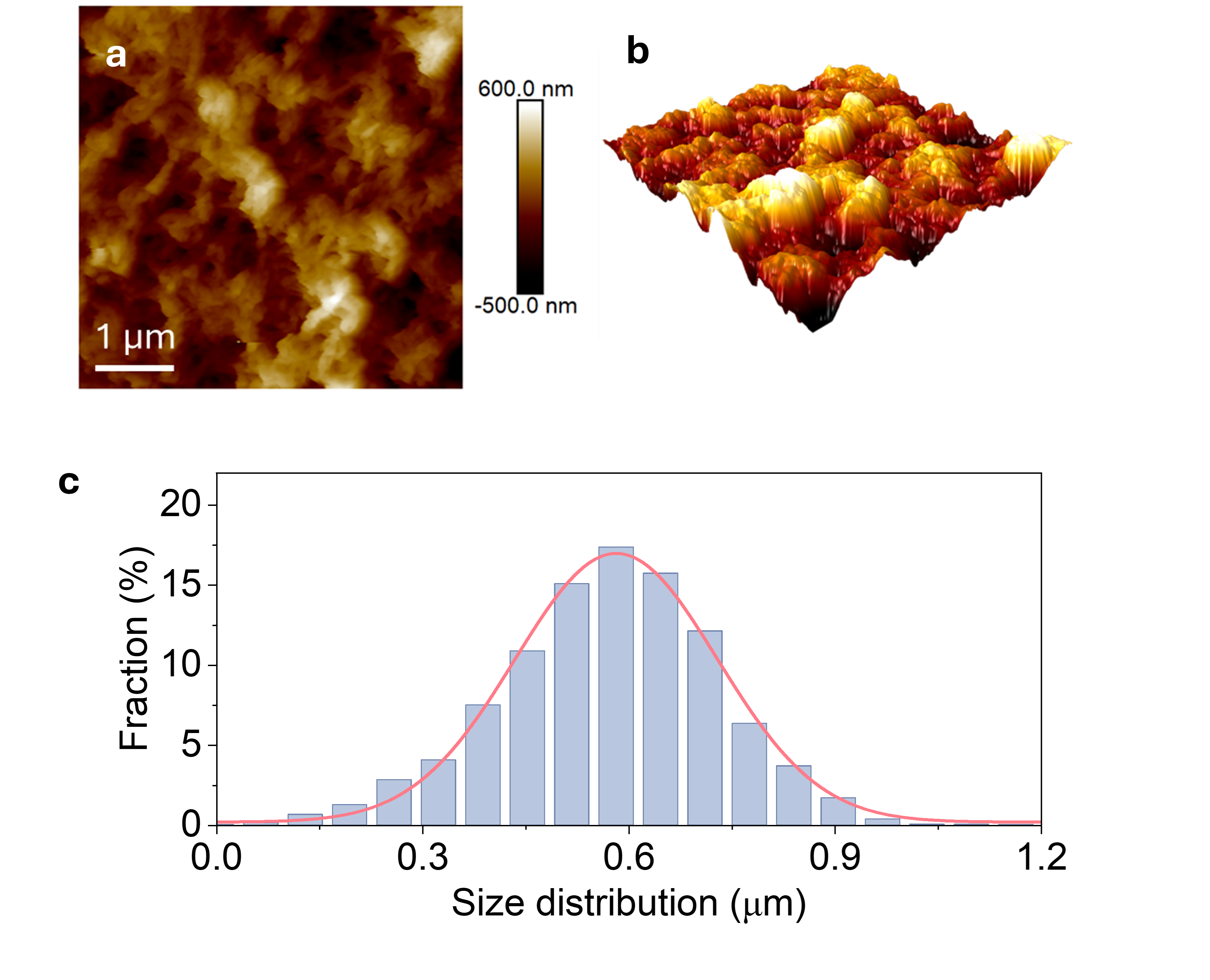


Figure S4. (a) Two-dimensional AFM topography image of the IP-COF membrane surface. (b) Three-dimensional AFM height map showing surface roughness features. (c) Height distribution histogram fitted with a Gaussian curve, indicating the average roughness of the IP-COF membrane.


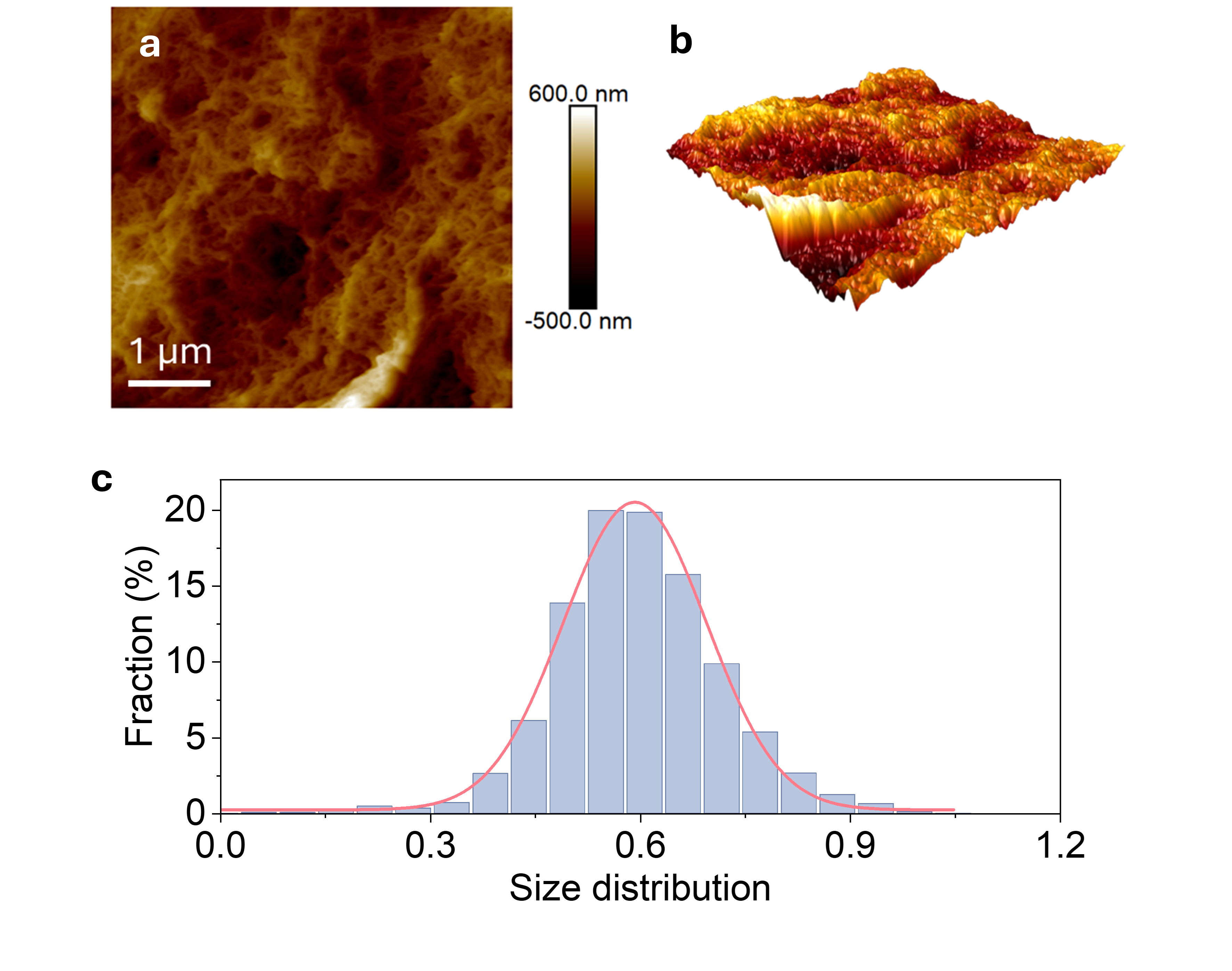


Figure S5. (a) Two-dimensional AFM topography image of the Healed-COF membrane surface. (b) Three-dimensional AFM height map showing surface roughness features. (c) Height distribution histogram fitted with a Gaussian curve, indicating the average roughness of the Healed-COF membrane.


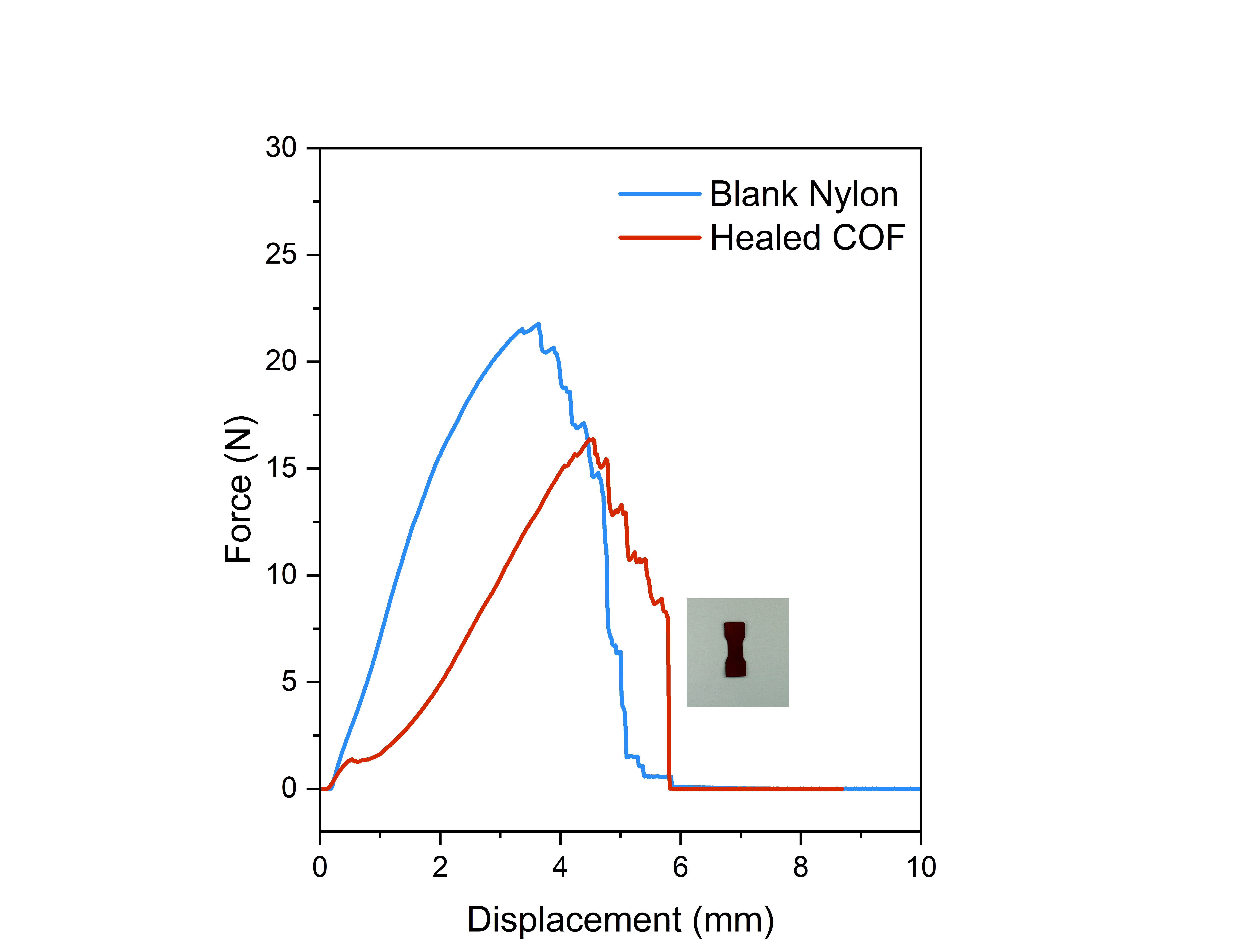


Figure S6. Force–displacement curves of blank nylon mesh and the healed COF membrane supported on nylon mesh.


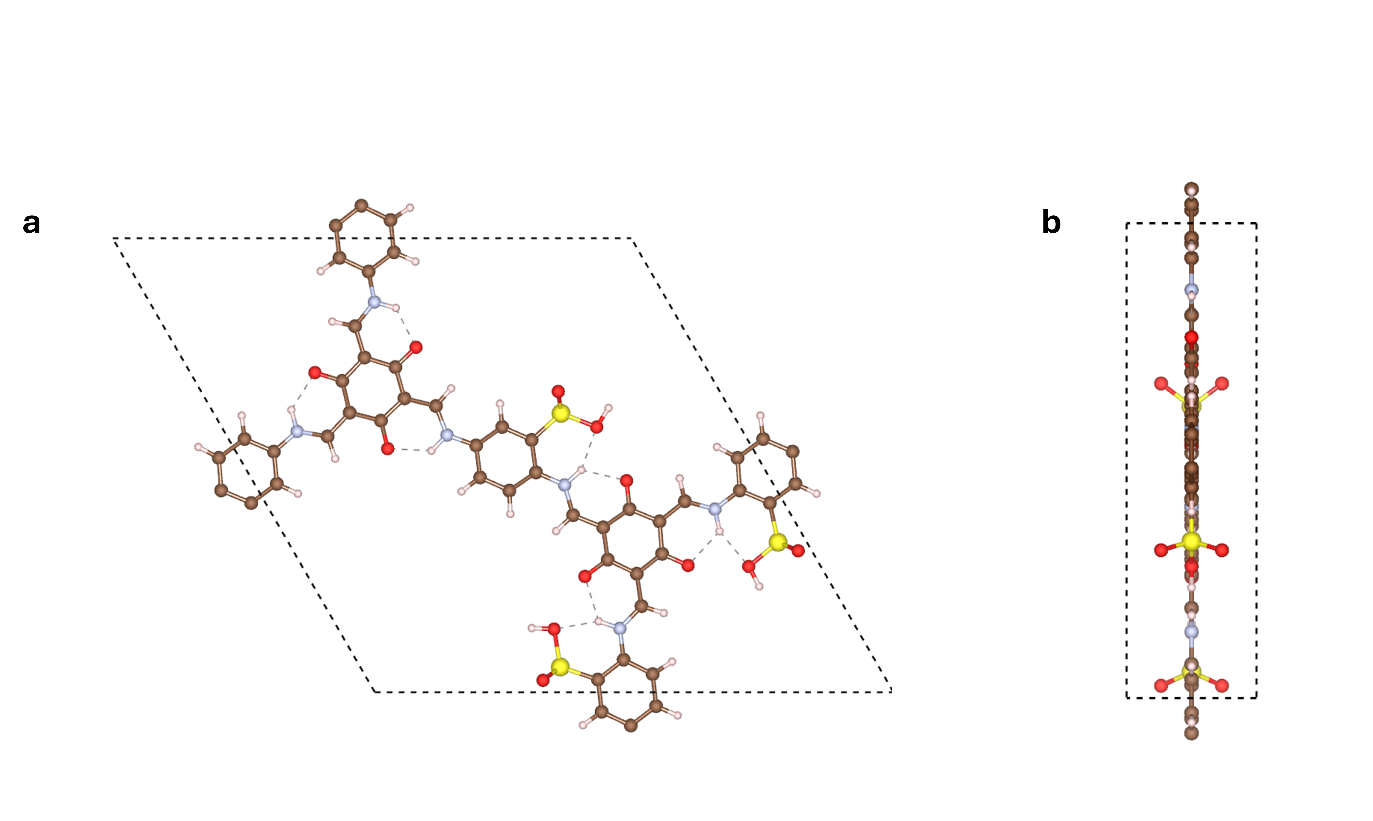


Figure S7. Schematic structural model of TpPa-SO₃H COF. (a) top view and (b) side view.


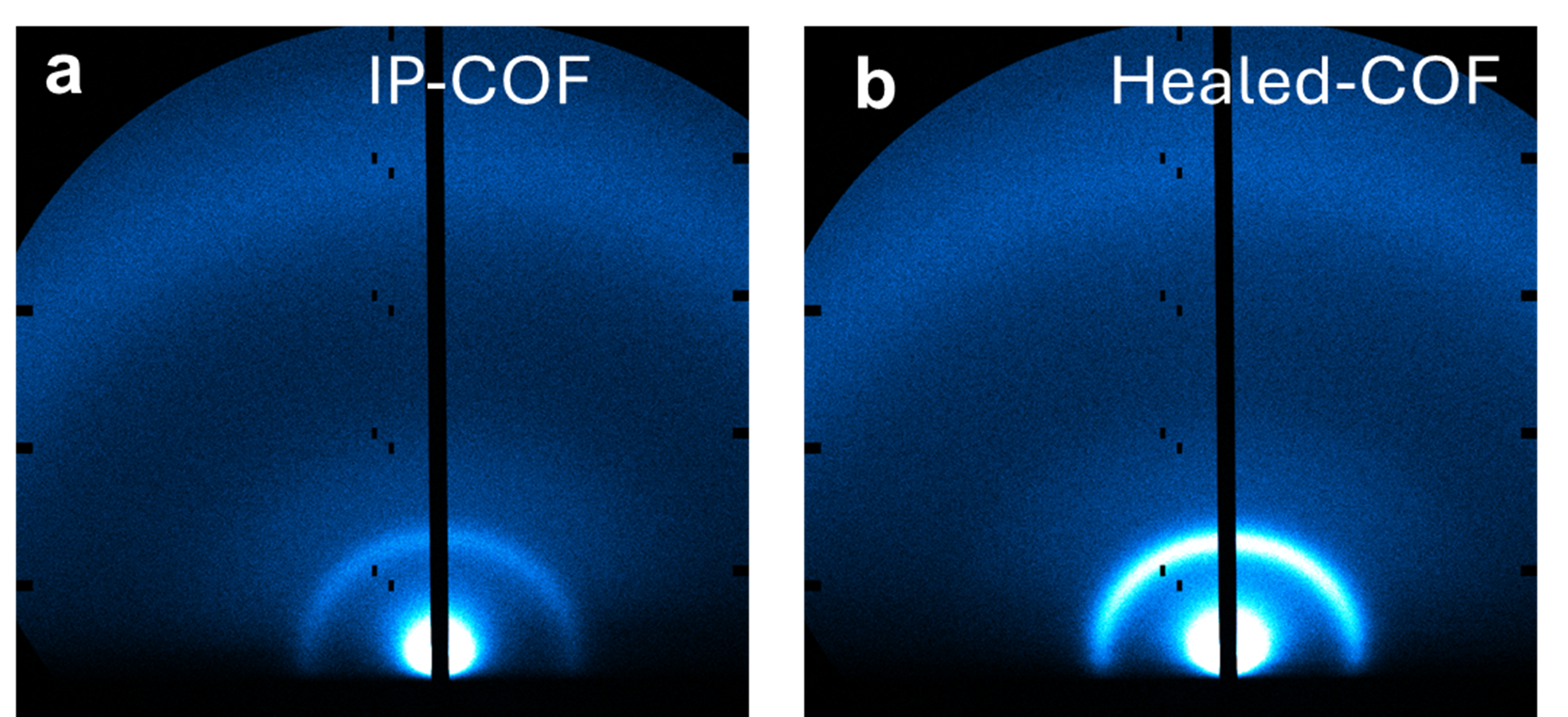


Figure S8. GISAX spectra of the (a) IP-COF membrane and (b) Healed-COF membrane.


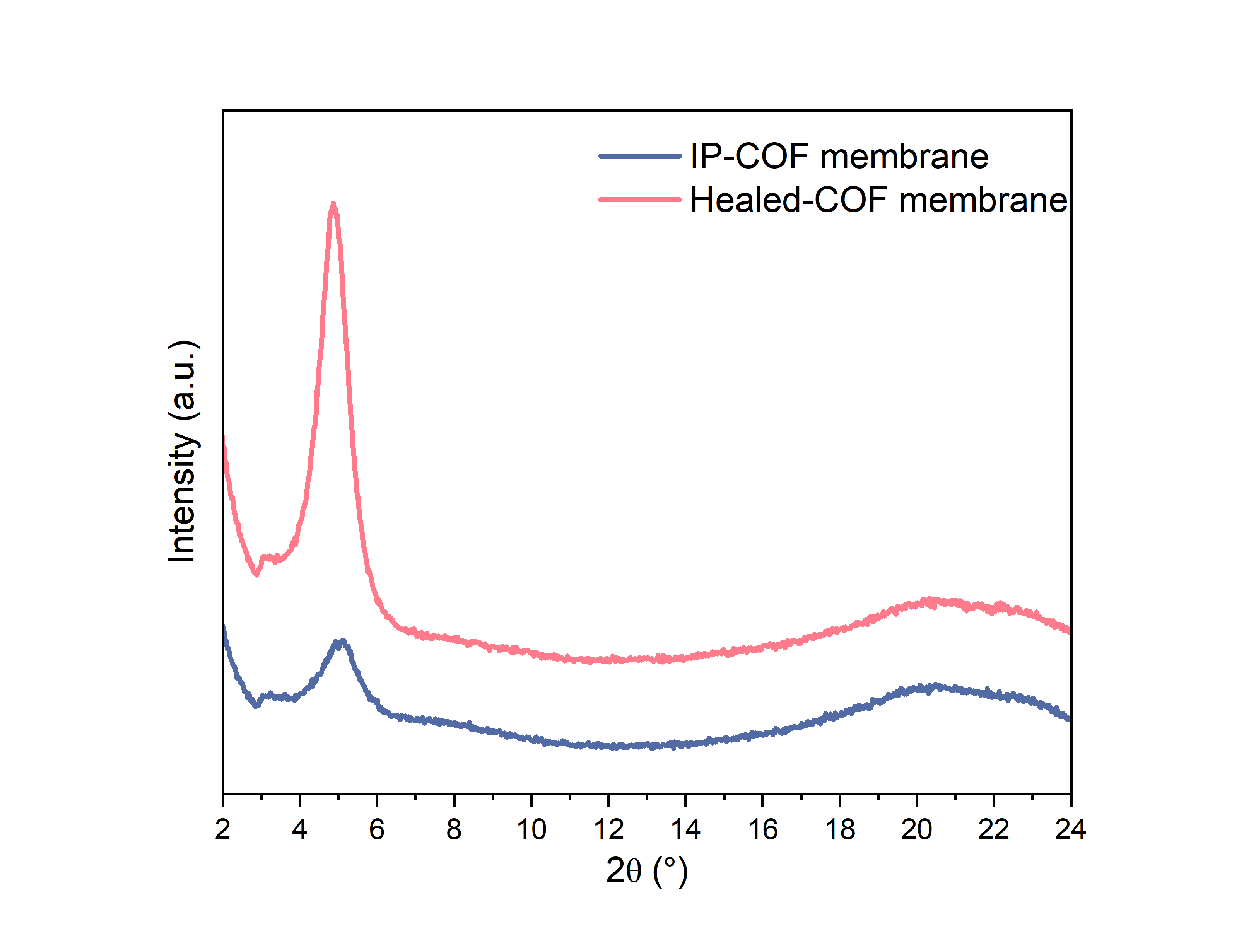


Figure S9. In-plane GIXRD patterns of the IP-COF membrane and Healed-COF membrane.


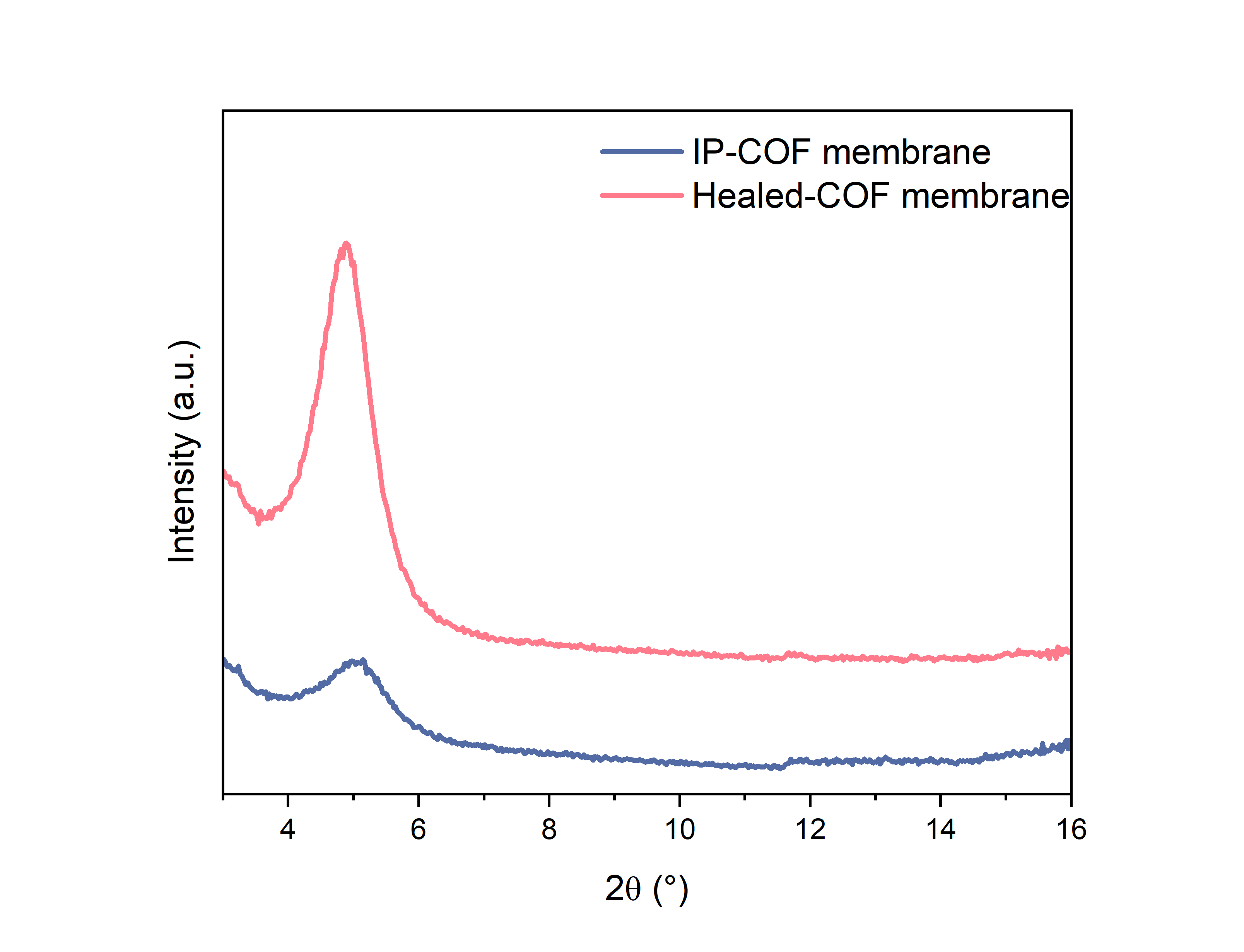


Figure S10. Out-plane GIXRD patterns of the IP-COF membrane (black) and Healed-COF membrane (red).

Figure S11. XRD spectra of the IP-COF membrane and solvent-treated-COF membrane. X-ray diffraction patterns from the as-prepared IP-COF and from a membrane subjected to 120 °C/72 h treatment in a mixed solvent (Dioxane and mesitylene, 4:1 v/v)^11, 12^ are shown. The solvent treatment was performed as a control to test whether simple thermal/solvent exposure or removal of loosely bound monomer/oligomer could account for the enhanced diffraction observed after acid-hydrothermal healing. No new reflections, peak shifts or systematic intensity changes beyond experimental scatter are apparent between the two traces, indicating that solvent swelling and thermal exposure alone do not produce the long-range lattice reordering observed after acid-catalyzed hydrothermal healing.


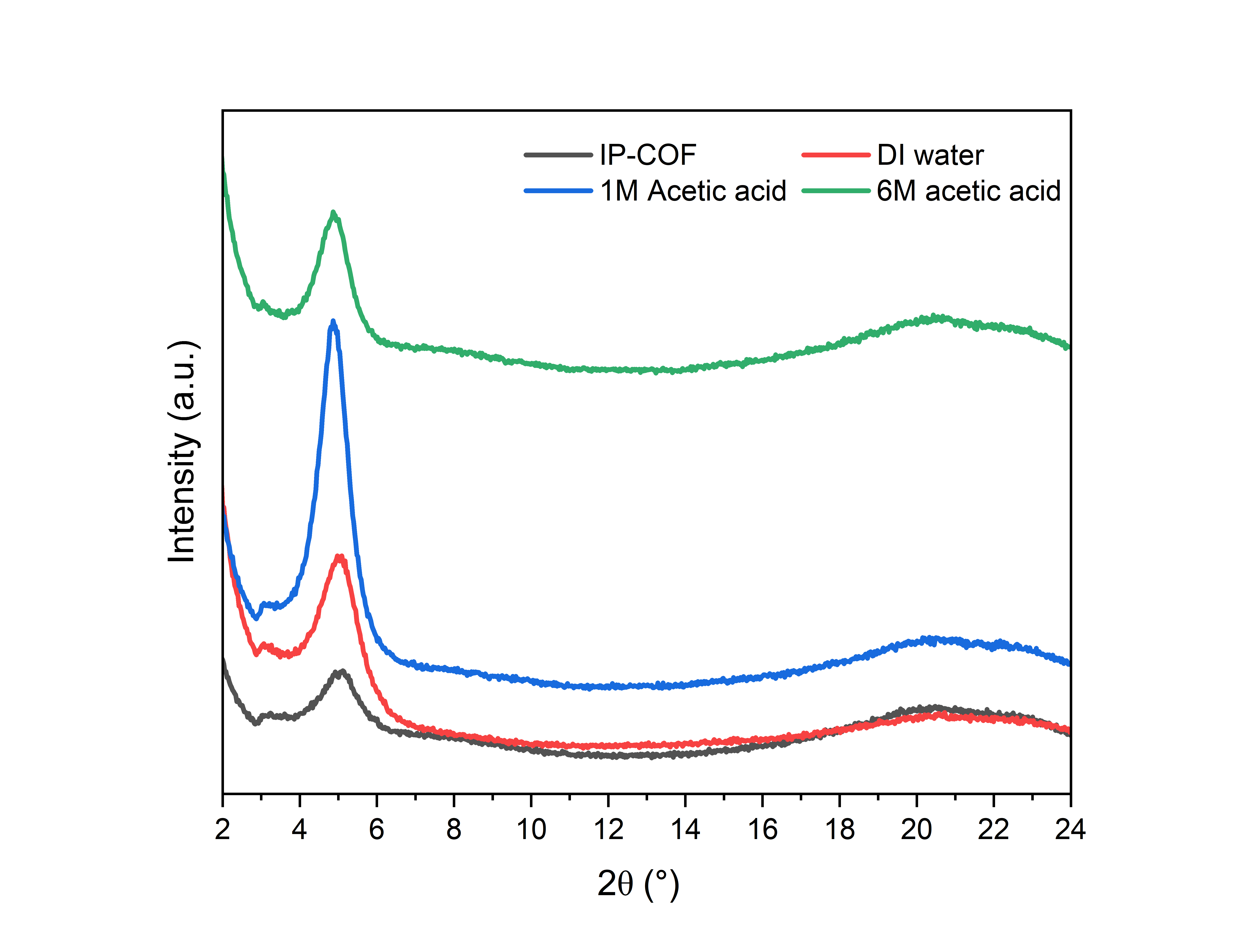
Figure S12. Comparison of the experimental XRD patterns treated with different solutions.

Figure S13. Simulated pore size distribution of AA-stacked TpPa-SO_3_H COF membrane.

Figure S14. Simulated pore size distribution of AB-stacked TpPa-SO_3_H COF membrane.


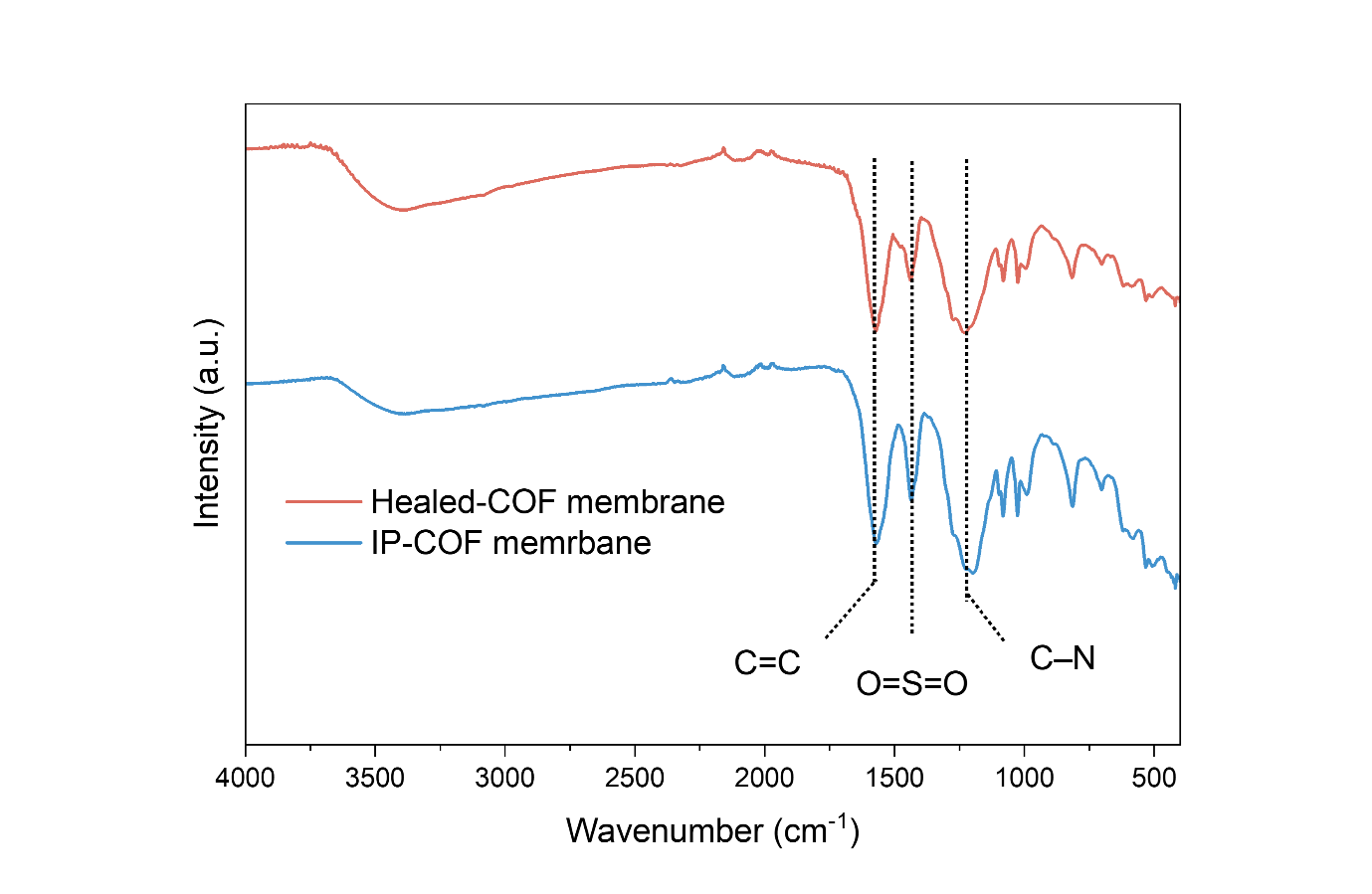


Figure S15. FTIR spectra of the IP-COF membrane and Healed-COF membrane.


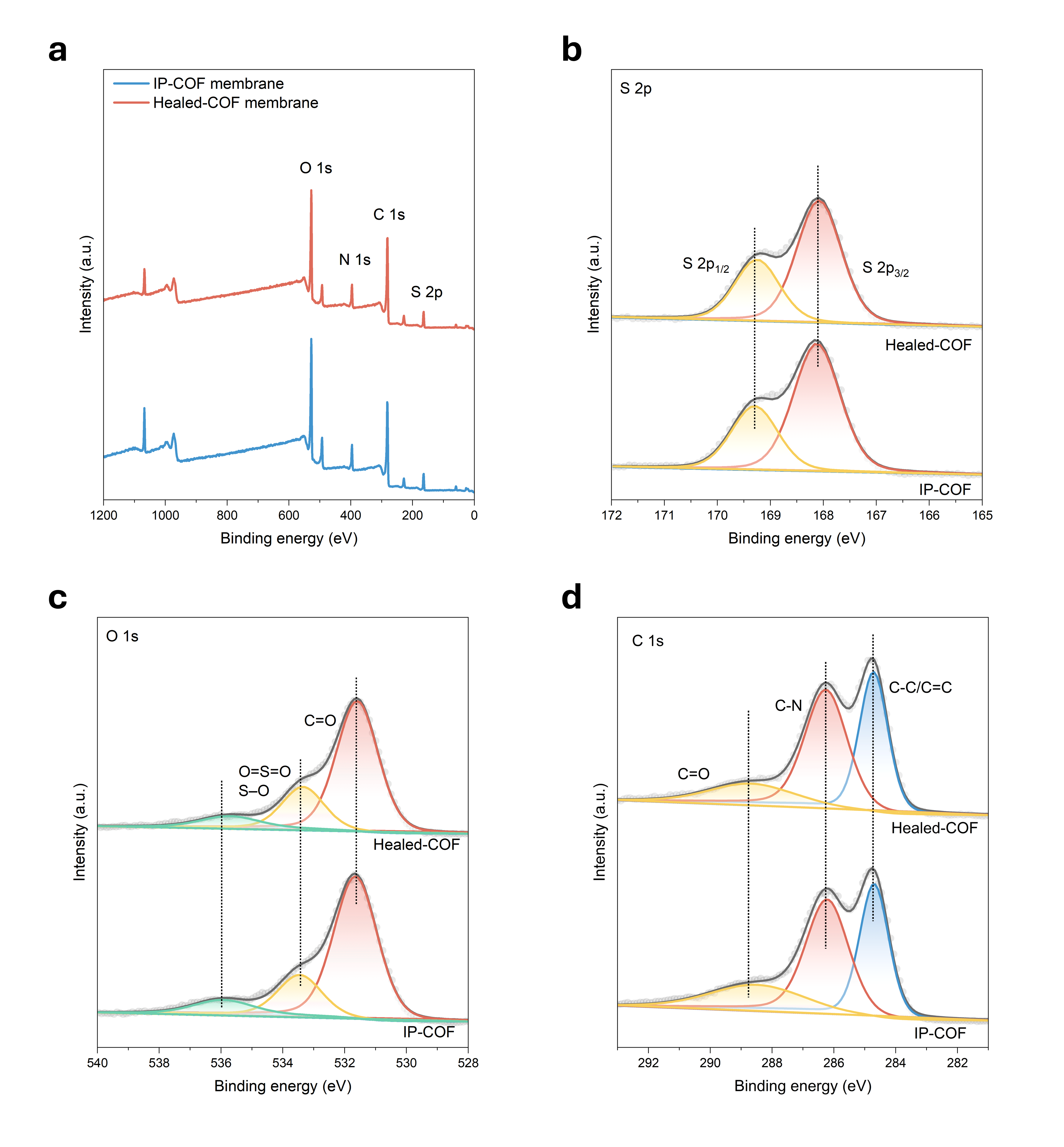


Figure S16. XPS survey spectrum of the IP-COF membrane and Healed-COF membrane, showing the presence of C, O, N, and S elements. (a) Survey spectra showing the overall elemental composition of IP-COF and healed COF membranes. High-resolution XPS spectra of S 2p (b), O 1s (c), and C 1s (d) regions with peak deconvolution, revealing the chemical states of sulphur, oxygen, and carbon species before and after healing.


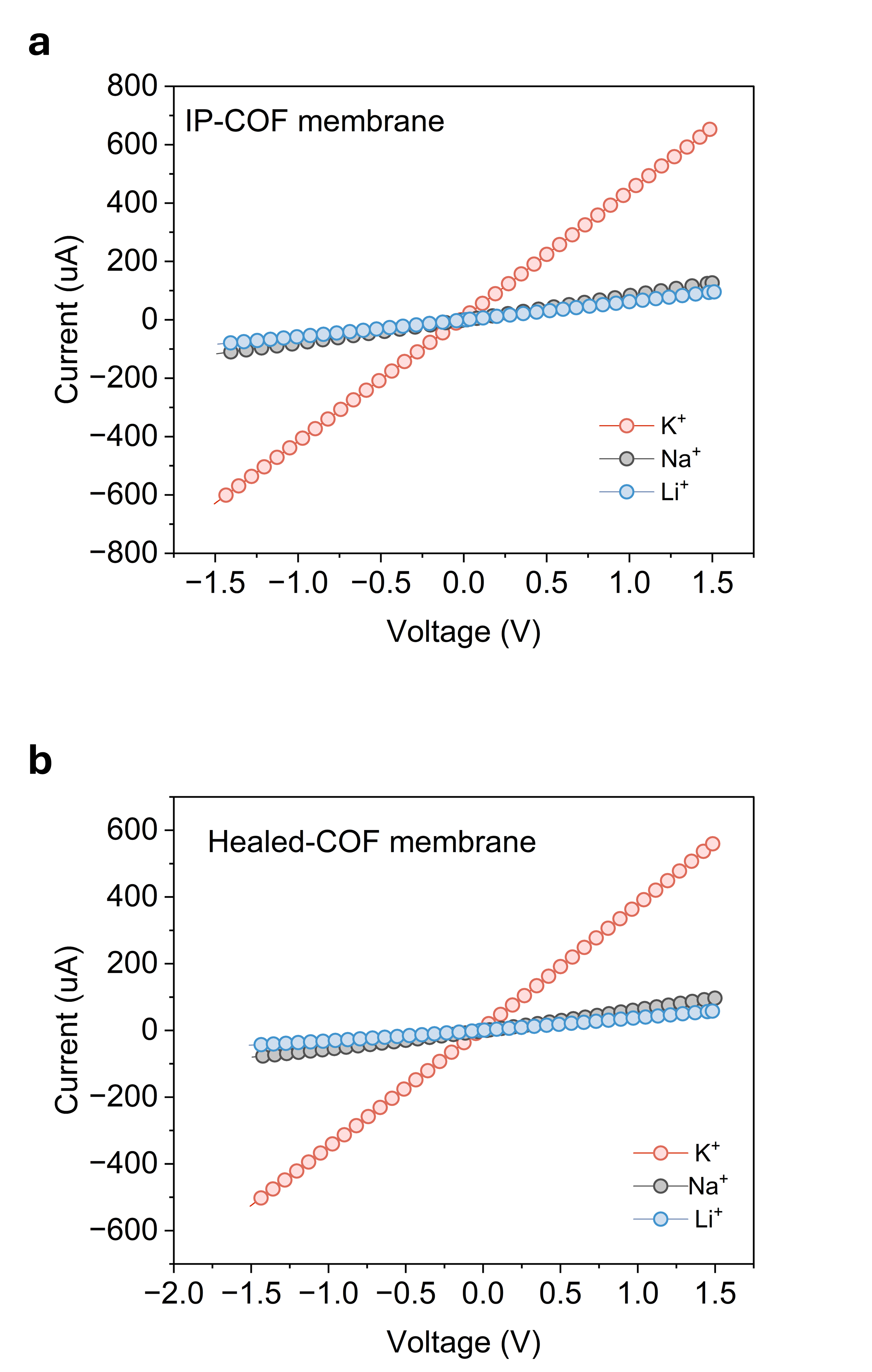


Figure S17. I-V curves of in different 100 mM electrolyte solutions of the IP-COF membrane (a) and Healed-COF membrane (b).


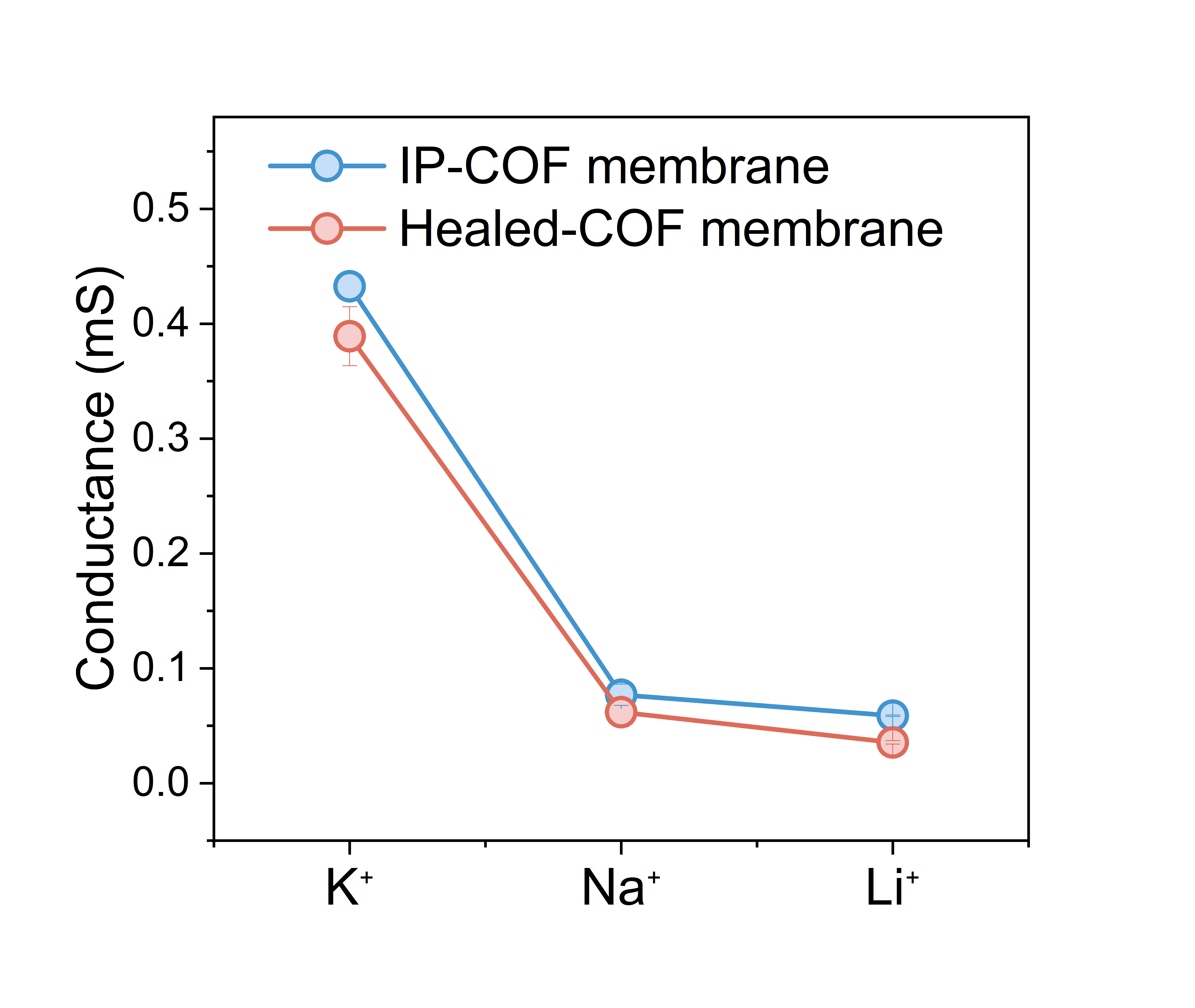


Figure S18.  Ionic conductance of the IP-COF membrane and Healed-COF membrane.


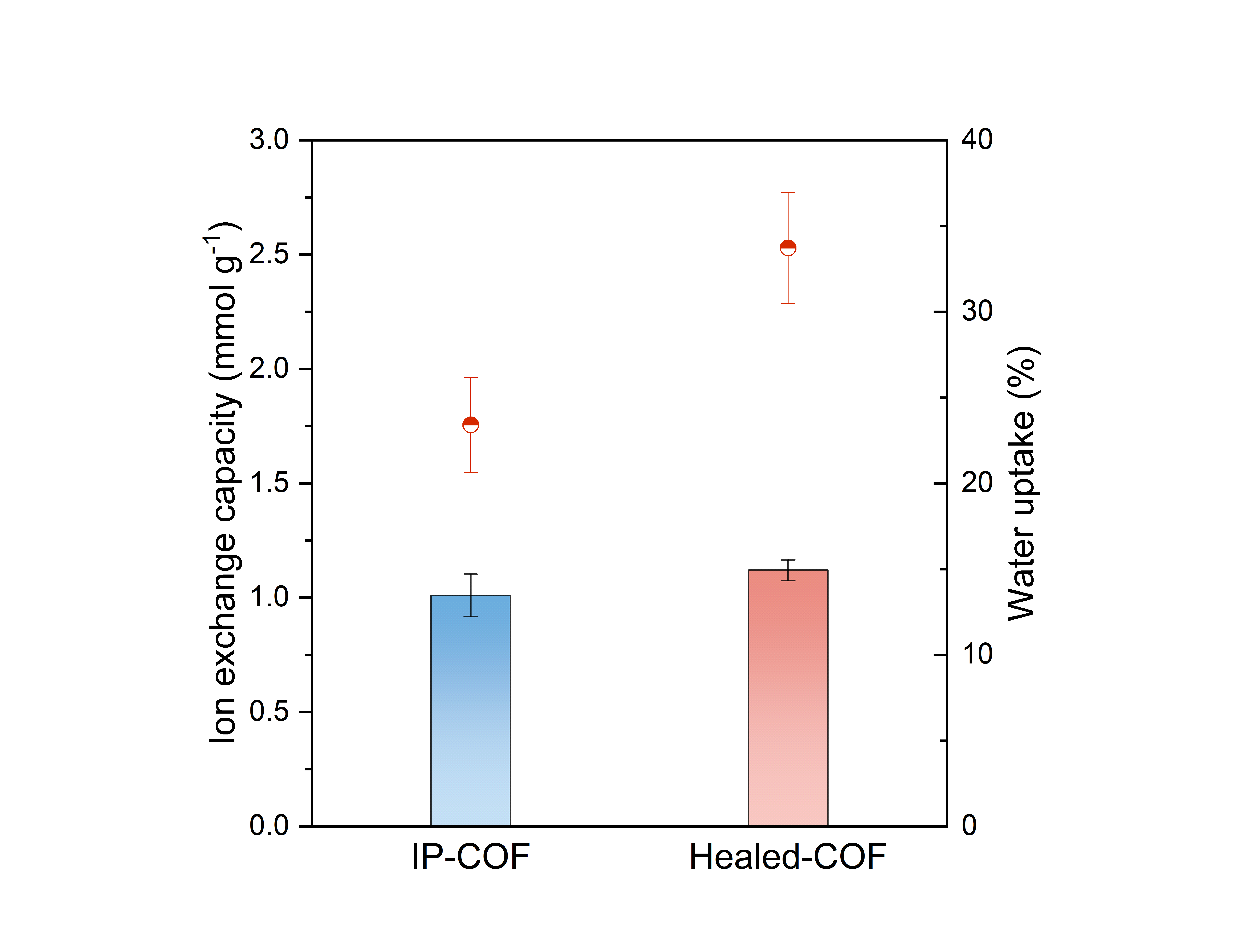


Figure S19. Comparison of ion exchange capacity (IEC, left axis) and water uptake (right axis) for IP-COF and Healed-COF membranes.


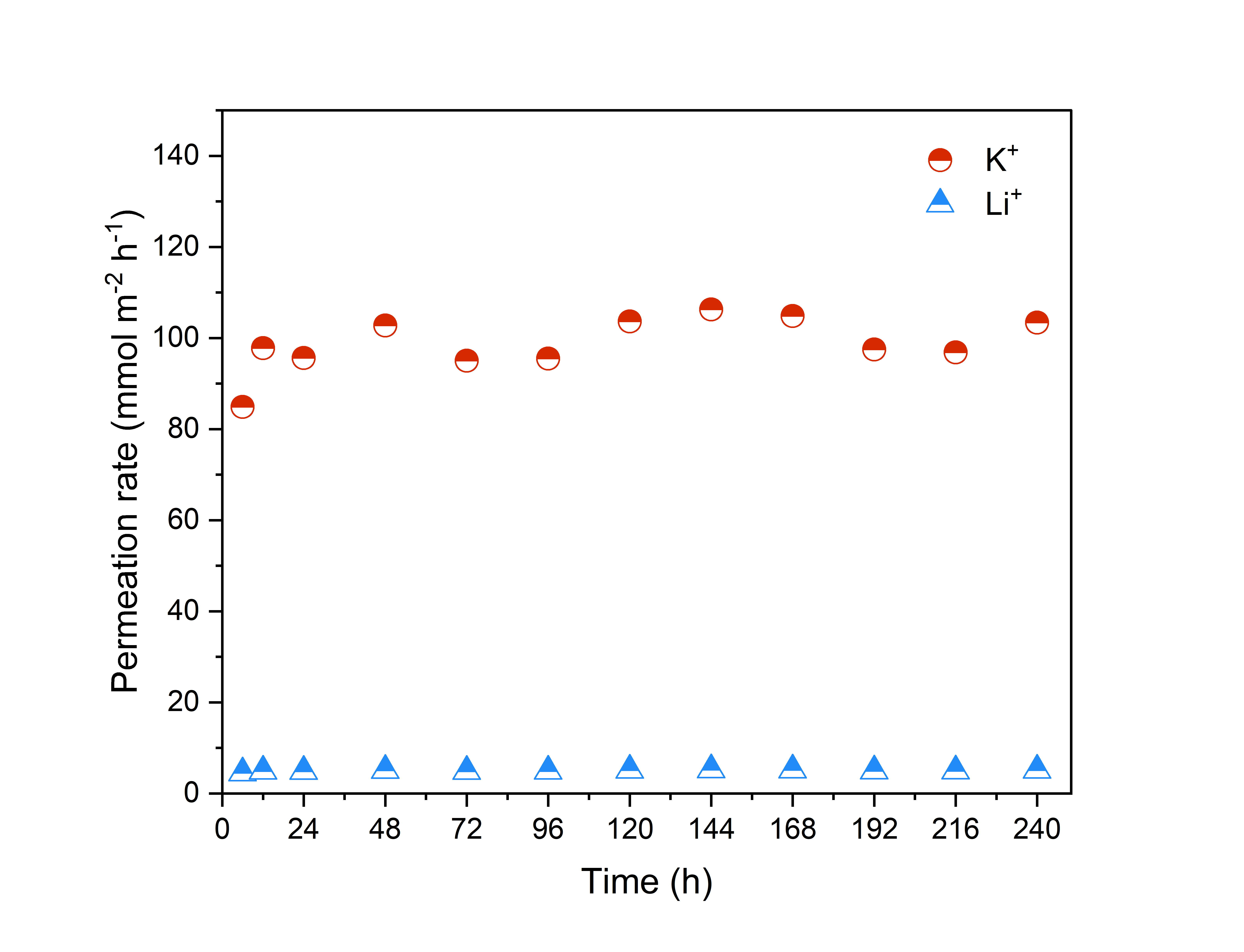
Figure S20. Time-dependent permeation rates of K^+^ and Li^+^ ions through the COF membrane measured over 240 h.

Table S1. Comparison of membranes for proton conductivity (T=293K, 100%RH).

| Membrane | Proton Conductivity (S cm^-1^) | Thickness  (um) | Ref. |
| --- | --- | --- | --- |
| Nafion 212 | 0.075 | 51 | ^13^ |
| SPEEK | 0.0179 | 60-70 | ^14^ |
| 3D SCOF | 0.27 | 1 | ^15^ |
| ZNUT-COF-SO_3_H | 0.1 | - | ^16^ |
| NUS-10(R) | 0.005 | 80 | ^17^ |
| IPC-COF | 0.15 | 1 | ^18^ |
| TpBD-(SO_3_H)_2_ | 0.23 | 3 | ^1^ |
| TpBD-C_4_- SO_3_H | 0.21 | 6-8 | ^19^ |
| SCON/SNF -4 | 0.2 | <1 | ^20^ |
| GO monolayer | 0.09 | <1 | ^21^ |
| SPEEK/abCOF | 0.12 | 20-30 | ^22^ |
| PA/PUNSNPs/SPEEK | 0.074 | - | ^23^ |
| m-HSbP_2_O_8_ | 0.04 | 15 | ^24^ |
| DNA@ZIF-8-3/25 | 0.005 | 0.5 | ^25^ |
| IP-COF | 0.02 | 0.74 | This work |
| Healed-COF | 0.11 | 0.74 | This work |

REFERENCE

(1) Wang, X.; Shi, B.; Yang, H.; Guan, J.; Liang, X.; Fan, C.; You, X.; Wang, Y.; Zhang, Z.; Wu, H.; et al. Assembling covalent organic framework membranes with superior ion exchange capacity. *Nature Communications* **2022**, *13* (1), 1020. DOI: 10.1038/s41467-022-28643-8.

(2) Wang, H.; Zhai, Y.; Li, Y.; Cao, Y.; Shi, B.; Li, R.; Zhu, Z.; Jiang, H.; Guo, Z.; Wang, M.; et al. Covalent organic framework membranes for efficient separation of monovalent cations. *Nature Communications* **2022**, *13* (1), 7123. DOI: 10.1038/s41467-022-34849-7.

(3) Zhu, D.; Verduzco, R. Ultralow Surface Tension Solvents Enable Facile COF Activation with Reduced Pore Collapse. *ACS Applied Materials & Interfaces* **2020**, *12* (29), 33121-33127. DOI: 10.1021/acsami.0c09173.

(4) Zhu, Q.; Shi, L.; Li, Z.; Li, G.; Xu, X. Protonation of an Imine-linked Covalent Organic Framework for Efficient H2O2 Photosynthesis under Visible Light up to 700 nm. *Angewandte Chemie International Edition* **2024**, *63* (32), e202408041. DOI: <https://doi.org/10.1002/anie.202408041>.

(5) Wang, Z.; Yang, J.; Yong, M.; Zeng, X.; Tebyetekerwa, M.; Sun, K.; Bie, C.; Xing, C.; Wang, H.; Andreeva, D. V.; et al. From Layered Crystals to Permselective Membranes: History, Fundamentals, and Opportunities. *Chemical Reviews* **2025**, *125* (14), 6753-6818. DOI: 10.1021/acs.chemrev.5c00025.

(6) Bessinger, D.; Ascherl, L.; Auras, F.; Bein, T. Spectrally Switchable Photodetection with Near-Infrared-Absorbing Covalent Organic Frameworks. *Journal of the American Chemical Society* **2017**, *139* (34), 12035-12042. DOI: 10.1021/jacs.7b06599.

(7) Zhang, G.; Tsujimoto, M.; Packwood, D.; Duong, N. T.; Nishiyama, Y.; Kadota, K.; Kitagawa, S.; Horike, S. Construction of a Hierarchical Architecture of Covalent Organic Frameworks via a Postsynthetic Approach. *Journal of the American Chemical Society* **2018**, *140* (7), 2602-2609. DOI: 10.1021/jacs.7b12350.

(8) Mayo, S. L.; Olafson, B. D.; Goddard, W. A. DREIDING: a generic force field for molecular simulations. *The Journal of Physical Chemistry* **1990**, *94* (26), 8897-8909. DOI: 10.1021/j100389a010.

(9) Rappe, A. K.; Goddard, W. A., III. Charge equilibration for molecular dynamics simulations. *The Journal of Physical Chemistry* **1991**, *95* (8), 3358-3363. DOI: 10.1021/j100161a070.

(10) Willems, T. F.; Rycroft, C. H.; Kazi, M.; Meza, J. C.; Haranczyk, M. Algorithms and tools for high-throughput geometry-based analysis of crystalline porous materials. *Microporous and Mesoporous Materials* **2012**, *149* (1), 134-141. DOI: <https://doi.org/10.1016/j.micromeso.2011.08.020>.

(11) Yang, J.; Acharjya, A.; Ye, M. Y.; Rabeah, J.; Li, S.; Kochovski, Z.; Youk, S.; Roeser, J.; Grüneberg, J.; Penschke, C. Protonated Imine‐Linked Covalent Organic Frameworks for Photocatalytic Hydrogen Evolution. *Angewandte Chemie International Edition* **2021**, *60* (36), 19797-19803.

(12) Zhu, D.; Zhu, Y.; Chen, Y.; Yan, Q.; Wu, H.; Liu, C.-Y.; Wang, X.; Alemany, L. B.; Gao, G.; Senftle, T. P. Three-dimensional covalent organic frameworks with pto and mhq-z topologies based on Tri-and tetratopic linkers. *Nature Communications* **2023**, *14* (1), 2865.

(13) Wang, H.; Li, X.; Zhuang, X.; Cheng, B.; Wang, W.; Kang, W.; Shi, L.; Li, H. Modification of Nafion membrane with biofunctional SiO2 nanofiber for proton exchange membrane fuel cells. *Journal of Power Sources* **2017**, *340*, 201-209. DOI: <https://doi.org/10.1016/j.jpowsour.2016.11.072>.

(14) Yin, Y.; Xu, T.; He, G.; Jiang, Z.; Wu, H. Fabrication of sulfonated poly(ether ether ketone)-based hybrid proton-conducting membranes containing carboxyl or amino acid-functionalized titania by in situ sol–gel process. *Journal of Power Sources* **2015**, *276*, 271-278. DOI: <https://doi.org/10.1016/j.jpowsour.2014.11.132>.

(15) Zhu, T.; Kong, Y.; Lyu, B.; Cao, L.; Shi, B.; Wang, X.; Pang, X.; Fan, C.; Yang, C.; Wu, H.; et al. 3D covalent organic framework membrane with fast and selective ion transport. *Nature Communications* **2023**, *14* (1), 5926. DOI: 10.1038/s41467-023-41555-5.

(16) Shao, Z.; Xue, X.; Gao, K.; Chen, J.; Zhai, L.; Wen, T.; Xiong, S.; Hou, H.; Mi, L. Sulfonated covalent organic framework packed Nafion membrane with high proton conductivity for H2/O2 fuel cell applications. *Journal of Materials Chemistry A* **2023**, *11* (7), 3446-3453, 10.1039/D2TA08435J. DOI: 10.1039/D2TA08435J.

(17) Peng, Y.; Xu, G.; Hu, Z.; Cheng, Y.; Chi, C.; Yuan, D.; Cheng, H.; Zhao, D. Mechanoassisted Synthesis of Sulfonated Covalent Organic Frameworks with High Intrinsic Proton Conductivity. *ACS Applied Materials & Interfaces* **2016**, *8* (28), 18505-18512. DOI: 10.1021/acsami.6b06189.

(18) Cao, L.; Wu, H.; Cao, Y.; Fan, C.; Zhao, R.; He, X.; Yang, P.; Shi, B.; You, X.; Jiang, Z. Weakly Humidity-Dependent Proton-Conducting COF Membranes. *Advanced Materials* **2020**, *32* (52), 2005565. DOI: <https://doi.org/10.1002/adma.202005565>.

(19) Shi, B.; Pang, X.; Lyu, B.; Wu, H.; Shen, J.; Guan, J.; Wang, X.; Fan, C.; Cao, L.; Zhu, T.; et al. Spacer-Engineered Ionic Channels in Covalent Organic Framework Membranes toward Ultrafast Proton Transport. *Advanced Materials* **2023**, *35* (16), 2211004. DOI: <https://doi.org/10.1002/adma.202211004>.

(20) Li, P.; Zhang, N.; Li, X.; Tang, S. Silk nanofibril as nanobinder for preparing COF nanosheet-based proton exchange membrane. *Green Energy & Environment* **2023**, *8* (3), 915-926. DOI: <https://doi.org/10.1016/j.gee.2022.05.008>.

(21) Wu, Z. F.; Sun, P. Z.; Wahab, O. J.; Tan, Y. T.; Barry, D.; Periyanagounder, D.; Pillai, P. B.; Dai, Q.; Xiong, W. Q.; Vega, L. F.; et al. Proton and molecular permeation through the basal plane of monolayer graphene oxide. *Nature Communications* **2023**, *14* (1), 7756. DOI: 10.1038/s41467-023-43637-w.

(22) Zhu, S.; Zhou, M.; Dong, X.; Wei, L.; Gao, Z.; Liu, Z.; Zhou, Z.; Wu, H.; Jiang, Z. Covalent organic frameworks with localized acid-base pairs confer enhanced proton conductivity. *Chemical Engineering Journal* **2025**, *523*, 168352. DOI: <https://doi.org/10.1016/j.cej.2025.168352>.

(23) Li, P.; Chen, Y.; Xiao, F.; Cao, M.; Pan, J.; Zheng, J.; Zhao, K.; Li, H.; Zhang, X.; Zhang, Y. An enhanced proton conductivity of proton exchange membranes by constructing proton-conducting nanopores using PVP-UiO-66-NH-SO3H nanoparticles. *International Journal of Hydrogen Energy* **2024**, *50*, 1020-1035. DOI: <https://doi.org/10.1016/j.ijhydene.2023.10.015>.

(24) Zhang, Z.; Liang, L.; Feng, J.; Hou, G.; Ren, W. Significant enhancement of proton conductivity in solid acid at the monolayer limit. *Nature Communications* **2024**, *15* (1), 2706. DOI: 10.1038/s41467-024-46911-7.

(25) Guo, Y.; Jiang, Z.; Ying, W.; Chen, L.; Liu, Y.; Wang, X.; Jiang, Z.-J.; Chen, B.; Peng, X. A DNA-Threaded ZIF-8 Membrane with High Proton Conductivity and Low Methanol Permeability. *Advanced Materials* **2018**, *30* (2), 1705155. DOI: <https://doi.org/10.1002/adma.201705155>.
